# Cationic Substitutions in Hydroxyapatite: Current Status of the Derived Biofunctional Effects and Their In Vitro Interrogation Methods

**DOI:** 10.3390/ma11112081

**Published:** 2018-10-24

**Authors:** Teddy Tite, Adrian-Claudiu Popa, Liliana Marinela Balescu, Iuliana Maria Bogdan, Iuliana Pasuk, José M. F. Ferreira, George E. Stan

**Affiliations:** 1 National Institute of Materials Physics, RO-077125 Magurele, Romania; teddy.tite@infim.ro (T.T.); adrian.claudiu@gmail.com (A.-C.P.); liliana.trinca@infim.ro (L.M.B.); iuliana.bogdan@infim.ro (I.M.B.); iuliana.pasuk@infim.ro (I.P.); 2 Army Centre for Medical Research, RO-010195 Bucharest, Romania; 3 Department of Materials and Ceramics Engineering, CICECO, University of Aveiro, 3810-193 Aveiro, Portugal

**Keywords:** biomedicine, hydroxyapatite, cation substitution, co-doping, biological assays

## Abstract

High-performance bioceramics are required for preventing failure and prolonging the life-time of bone grafting scaffolds and osseous implants. The proper identification and development of materials with extended functionalities addressing socio-economic needs and health problems constitute important and critical steps at the heart of clinical research. Recent findings in the realm of ion-substituted hydroxyapatite (HA) could pave the road towards significant developments in biomedicine, with an emphasis on a new generation of orthopaedic and dentistry applications, since such bioceramics are able to mimic the structural, compositional and mechanical properties of the bone mineral phase. In fact, the fascinating ability of the HA crystalline lattice to allow for the substitution of calcium ions with a plethora of cationic species has been widely explored in the recent period, with consequent modifications of its physical and chemical features, as well as its functional mechanical and in vitro and in vivo biological performance. A comprehensive inventory of the progresses achieved so far is both opportune and of paramount importance, in order to not only gather and summarize information, but to also allow fellow researchers to compare with ease and filter the best solutions for the cation substitution of HA-based materials and enable the development of multi-functional biomedical designs. The review surveys preparation and synthesis methods, pinpoints all the explored cation dopants, and discloses the full application range of substituted HA. Special attention is dedicated to the antimicrobial efficiency spectrum and cytotoxic trade-off concentration values for various cell lines, highlighting new prophylactic routes for the prevention of implant failure. Importantly, the current in vitro biological tests (widely employed to unveil the biological performance of HA-based materials), and their ability to mimic the in vivo biological interactions, are also critically assessed. Future perspectives are discussed, and a series of recommendations are underlined.

## 1. Introduction

Over the last decades, the biomedical orthopaedic and dentistry sectors have witnessed an unprecedented demand for a large variety and number of scaffolds, grafts, implants, and endo-prostheses. The increase in life expectancy, and the higher frequency of injuries and diseases are regarded as the main factors for this growing demand in orthopaedic and dental devices. The quality of life for millions of people has been drastically improved by using hydroxyapatite (HA) and bioactive glasses (BGs) for bone repair and tissue regeneration [1,2,3,4,5,6,7,8,9,10,11,12,13,14,15,16,17,18,19]. In particular, synthetic stoichiometric hydroxyapatite (having the Ca_10_(PO_4_)_6_(OH)_2_ stoichiometry and theoretical Ca/P molar ratio of 1.67), calcium-deficient hydroxyapatite, or oxyapatite have been widely used as prominent bioactive materials in healthcare due to their excellent biocompatibility, non-toxicity and osteoconductive properties [1,2,20,21,22]. This was an obvious choice, since these materials are inspired by nature, HA being the major mineral component of hard conjunctive tissues (e.g., bone and teeth). Moreover, HA is able to accommodate healthy osteoblast (bone growth cells) and osteoclast (bone resorption cells) adhesion and growth, as well as to promote stem cell proliferation and differentiation [1,2,23,24,25,26]. However, synthetic HA has poor mechanical properties (e.g., poor tensile strength and low fracture toughness), and cannot be used for the fabrication of mechanically safe load-bearing bulk implants or prostheses. Thereby, HA use is limited to porous scaffolds as bone grafts or fillers [27,28,29,30,31] and coatings for the bio-functionalization of metallic implants [1,2,23,32,33,34,35,36,37,38,39].

Studies concerning the electrical properties of HA occupy a special place in the research topic of biomaterials. The discovery of the piezoelectric effect in dry bone by Fukada and Yasuda in 1957 [40] seemed to offer an explanation to certain observed phenomena, such as bone remodelling as the effect of electric charge accumulation on the surface of crystallites due to mechanical stress. Fast bone fracture healing was observed when mechanical loading was applied [41,42]. The process of mechano-transduction was advanced as a possible cause for osteogenesis [41]. It is believed that the electrical dipoles generated by the action of mechanical stress on the bone matrix (collagen and hydroxyapatite exhibiting piezoelectric properties) could increase the interaction with the cells pointing them in the direction of “force lines” (for the production of 3D tracts with a 3D disposition that supports maximum mechanical stress) and could attract calcium and phosphate ions, leading to the acceleration of mineralization, regeneration and bone growth processes. Also, the mechanical stress activates specific adaptive responses in osteoblasts and osteoclast cells, their cytoskeleton being connected to the bone matrix through cell-matrix junctions. The actin cytoskeleton receives mechanical stress stimuli from the focal adhesion type of junction, which will activate the YAP (yes-associated protein 1) transcription co-activator and TEAD (transcriptional enhanced associate domain) transcription factor (through Hippo signalling pathway). This increases transduction of genes related to proliferation or differentiation (e.g., c-fos, egr-1, iex-1, c-myc) [43]. The protein p130Cas, a component of the focal adhesion junction acts also as a mechanical transduction molecule, leading to Rac activation. Another mechanism of cytoskeleton modulation of cellular function is performed through Rho-family GTPases. The microtubule and intermediate filaments of the cytoskeleton respond to mechanical stimuli by spatial migration and cell division direction in the tissue [43].

However, HA seemingly crystallizes in hexagonal symmetry, space group P6_3_/m, with an inversion centre, which does not allow the piezoelectric effect to occur. In the HA crystal lattice, the (PO_4_)^3−^ tetrahedrons are joined together by Ca^2+^ bridges. The space between (PO_4_)^3−^ groups is relatively large allowing the accommodation of foreign atoms with quite different diameters from Ca^2+^. The (OH)^−^ ions are aligned along the six-fold axis of the lattice, bounded by columns of Ca^2+^ and (PO_4_)^3−^ forming the so-called “apatitic channel”. Since the (OH)^−^ ions appear to play an important role in ionic conduction [44,45], a HA crystallite can be regarded as a one-dimensional anionic conductor along the *c*-axis [44,46].

Given the non-polar crystal structure of HA, Fukada and Yasuda [40] attributed the observed piezoelectric effect to collagen, a protein of quasi-ordered, polar structure, which definitely has piezoelectric behaviour. Although most researchers in the field agree that the responsibility for the clearly proven piezoelectricity of dry bones is the collagen structure, there are still groups that associate (at least partially) the phenomenon to the intrinsic structure of mineral HA. A possible alternative explanation would be that HA nanocrystals actually have a monoclinic, polar structure—space group P2_1_/b [47,48,49]. Since the monoclinic deformation is weak, the HA-monoclinic structure cannot be practically distinguished from the hexagonal one by powder diffractometry. Lang et al. [47,48,49] have obtained clear evidence of piezo-, pyro- [48] and even ferro-electric [49] behaviour of synthetic HA films (having a (001) preferential orientation of the 70–100 nm large crystallites) deposited by sol-gel on silicon substrate. The piezoelectric effect was also measured in HA ceramics sintered by spark plasma sintering [47]. Furthermore, Lang et al. stressed that not all HA particles give the equally strong piezoelectric signal, which is why the macroscopic effect is not conclusive [49].

**Figure 1:**
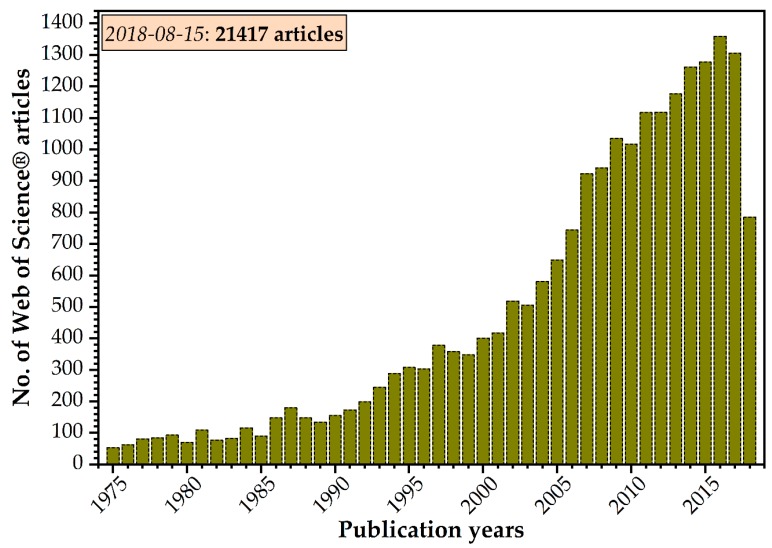
Yearly distribution of scientific articles published on the hydroxyapatite topic over the 1975–2018 period (15th of August 2018). Database: Clarivate Analytics—Web of Science^®^ Core Collection. Coupled «title» and «topic» search keywords: “hydroxyapatite”, “hydroxylapatite”, “HA”, “HAp”, “Ca_10_(PO_4_)_6_(OH)_2_, and “Ca_5_(PO_4_)_3_(OH)”.

The continuous effervescence of the ever-topical HA research field is demonstrated by a progressive yearly increase of published papers (Figure 1), since 2009 constantly exceeding more than 1000 items per year. To date more than 21,000 HA papers were published.

Biological apatites (bioapatites) are carbonated non-stoichiometric Ca-deficient compounds, substituted with trace amounts of various ions, either adsorbed on the crystal surface or incorporated in the lattice structure [1,2,5,32,50,51]. As a constituent of bones, HA also contains F which partially substitutes the hydroxyl group, and impurities such as Mg, Na or Si. Metals usually substitute one of the two crystallographic positions of Ca, while Si substitutes P. The valence differences between the host and substitute atoms are usually compensated by oxygen defects. In addition, the natural bone consists of carbonated HA, with the carbonate group replacing either the hydroxyl group—in this case the carbonated structure is called type-A, or the phosphate group—denoted as type-B structure [52]. These two types of carbonated HA structures were intensively studied by different structural methods, but reliable results were obtained only on synthetic HA, since rigorous structural studies (e.g., by diffraction on single crystals) on natural HA are limited because of the very small dimensions of bone crystallites [46]. Typically, the type-A or type-B HA structures are verified by Fourier transform infrared (FTIR) spectrometry by the identification of the positions of the ν_2_ and ν_3_ stretching vibration modes of the carbonate groups [53]. The mineral bone substitutions with trace elements are considered reasons for the changes in crystallinity, solubility, and the overall biological responses.

Besides biological structures/systems, the mineral named apatite—Ca_10_(PO_4_)_6_X_2_, X = F^−^, Cl^−^ or (OH)^−^ is an important host material for rare earth elements, often present as substituent for Ca. The crystal symmetry of these minerals is hexagonal with space group P63/m, or, for some ordered varieties, monoclinic with space group P21/b [54].

The limitation in terms of autografts and allografts has led scientists to the development of various non-synthetic (e.g., natural resources: animal, aquatic, plants) [21,22,55,56,57,58,59,60,61,62,63,64] and synthetic doped/substituted HA as alternatives [5,65,66,67,68]. The first type of “doping” involves cationic substitution, where monovalent (e.g., Ag^+^, Na^+^, K^+^), bivalent (e.g., Mg^2+^, Sr^2+^, Zn^2+^, Ba^2+^), or multi-valent cations move in the lattice sites occupied by Ca^2+^. A second type of doping implies the anionic substitution, occurring either at the hydroxyl site (type-A substitution), at the phosphate site (type-B substitution), or as a mixture of both (type-AB substitution).

**Figure 2:**
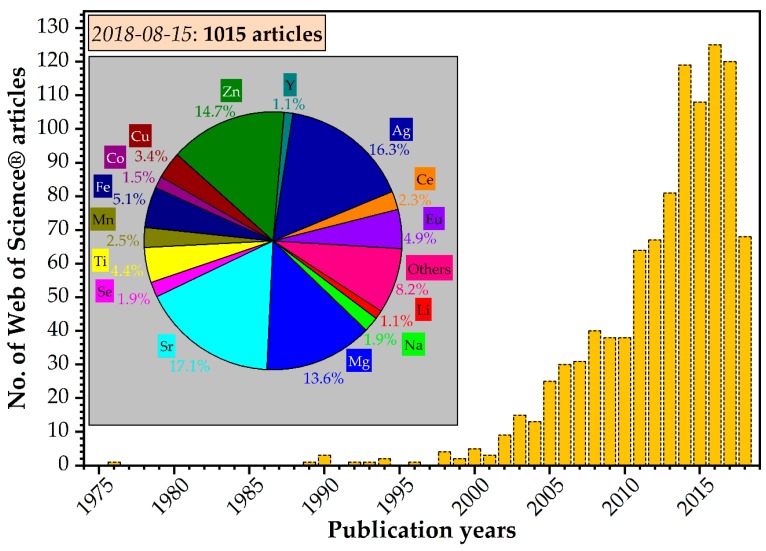
Yearly distribution of scientific articles published on the doped-substituted hydroxyapatite topic over the 1975–2018 period (15th of August 2018). Database: Clarivate Analytics—Web of Science^®^ Core Collection. Coupled «title» and «topic» search keywords: “hydroxyapatite”, “hydroxylapatite”, “HA”, “HAp”, “Ca_10_(PO_4_)_6_(OH)_2_”, “Ca_5_(PO_4_)_3_(OH)”, “doped”, and “substituted”. Inset: Frequency of cation doping in hydroxyapatite.

Important research activities have been devoted to substituted HA (SHA) compounds [1,2,32,35,69,70], and a large variety of cation doped/substituted hydroxyapatite materials have been synthesized (Figure 2). The most intensively studied doping cations were Sr, Ag, Zn, and Mg, with frequencies of ~17.5%, ~16.5%, ~15%, and~13.9%, respectively (Figure 2—inset).

The great variety of cation doping possibilities, of different ionic radii, is determined by the high lattice “flexibility”, and good structural stability of hydroxyapatite [5,71,72]. Synthetically substituted HAs seem to possess a series of significant advantages over stoichiometric HA, imprinted by the structural (e.g., changes in the lattice constants and unit cell volume, generation of defects, particular surface charge distributions) and morphological modifications the cation doping is inducing [1,2,32,35,69,73,74,75,76]. For instance, the fracture toughness (K_Ic_) is a decisive material property when developing reliable ceramic scaffold grafts for load-bearing applications. However, pure HA is a quite brittle material, with a K_Ic_ value situated in the range of 0.5–1 MPa·m^1/2^ [77], thus much lower than the cortical human bone which elicits K_Ic_ performances in the range 2–12 MPa·m^1/2^, depending on the direction of the applied mechanical load [75,77,78]. However, few reports have already hinted at the possibility of improving this specific mechanical property by controlled cation substitution in the HA lattice. Specifically, a K_Ic_ value of 2.7 MPa·m^1/2^ was achieved by doping HA with 0.6 wt.% Mg [79]. S. Lala et al. [80] reported a fracture toughness improvement from ~0.5 MPa·m^1/2^ (in the case of pure HA) to values of 1.0, 1.5, and 1.6 MPa·m^1/2^ when doping with 5 at.% Mg, Zn and Mn, respectively. Nonetheless, the data on the fracture toughness of doped HA are not abundant, and thereby further unambiguous explorations are needed to confirm or advance new doping possibilities able to improve the mechanical properties of HA, allowing for its safe use for load-bearing biomedical applications. Furthermore, the cation doping of HA started to be recognized for its ability to lead to improved biological properties such as bioactivity, surface reactivity, and adsorption of proteins/growth factors, while fostering biocompatibility, non-genotoxicity and ability to promote cell proliferation [1,2,5,32,69,73,74,81].

As the overall biological performance of bone regeneration substitutes and implants relies on positive interfacial interaction with body media and the surrounding tissues immediately after the implantation, the biofunctional advances introduced by cation substitution of HA could prove of high significance for the biomedical field, and need to be emphasized, as they could pave the road toward an upgraded generation of bioceramic scaffold and implant coatings.

Moreover, the concerns generated by both the (*i*) appearance of microorganisms resistant to all known pharmaceutical antibiotics [82,83] and (*ii*) increase of implant failure due to nosocomial infections with various pathogens [84], have lately driven the research into “equipping” the hydroxyapatite materials (by doping) also with antimicrobial defence mechanisms, with promising prospects, as will be shown in this review.

Thereby, justified by the ease of doping and the potential great biomedical benefits which could emerge from such scientific endeavours, hyper-active research has been dedicated to the cationic doping of HA, with the table of Elements serving as a playground for selecting either rational (based on the human bone trace elements) or exotic (e.g., lanthanides and actinides) cation dopants. 

This review focused on the current status of the cationic-substituted HA materials, their derived and many times complementary biofunctional effects (with deep regard on their cytotoxicity and antimicrobial activity), as well as critically surveying the most used in vitro biological interrogation/investigation assays and conceptual experimental designs, along with their ability to mimic the in vivo biological interactions. Conclusions will be drawn, future perspectives will be discussed, and a series of recommendations will be highlighted.

## 2. Preparation Methods and Synthesis Routes of Hydroxyapatite Materials

Bulk HA can be produced in various forms (e.g., nano-crystalline and micro-crystalline powders, granules, coatings) and shapes (e.g., spheres, platelets, needles, rods) from either synthetic or non-synthetic (natural) resources, both having their advantages and limitations.

For example, the methods used to extract/prepare HA from natural (sustainable/renewable) resources are cheap and simple (in the case of bone waste). Furthermore, the as-fabricated HA materials are well-suited to achieve a good synergy with biological media since they already contain trace element substitutions [22,50,85,86,87,88]. However, such materials are dependent on the availability of natural resources and require a well-controlled calcination procedures and cleaning protocols to remove the organic moieties, and most importantly any bacteria or viruses [22,89,90,91]. A large palette of natural HA sources including animal bones (mammalian, fish), plants, and biogenic (egg-shell and sea-shell) sources have been employed in order to respond to escalating orthopaedic and dentistry demands [22,55]. It has been argued that HA obtained from natural resources exhibits properties and a biological response comparable or even better than the synthetic ones, due to their similitude with bone apatite [92]. HA prepared from natural resources such as bovine, sheep, pig, fish, egg-shell, sea-shell or marble contain trace amounts of cations such as Na^+^, K^+^, Mg^2+^, Zn^2+^, Ba^2+^, or Sr^2+^ and anions such as CO_3_^2−^, or SiO_4_^4−^, F^−^ or Cl^−^, which play crucial roles in biochemical interactions, bone regeneration and tissue engineering [55].

### 2.1. Preparation of Bulk Hydroxyapatite (HA) from Natural Resources

HA has been prepared from various animal-origin sources including bovines [57,58,89,93,94,95,96,97,98], pigs [99,100,101], camels [102], sheep [103,104,105], goats [94], and chickens [94,106,107]. A summary of the frequently utilized natural resources for the synthesis of hydroxyapatite materials is presented in Table 1.

Bovine and swine bones seem to be the preferred animal resources for biological HA production [22,57,58,89,96,97,114]. Animal bone-derived HA is typically prepared by a three-stage process: (*i*) mechanical scraping of soft tissue; (*ii*) deproteinization in alkali media; (*iii*) calcination at temperatures able to remove any remnant organic and biological hazardous components. HA derived from animal bone has generally a low degree of crystallinity, and usually calcination between 600 and 1100 °C has been found to be the best choice to improve the degree of crystallinity with excellent thermal phase stability [55,88]. Rincón-López et al. [92] compared the physical and chemical properties of cortical bovine HA (BHA) with a commercial synthetic HA and observed that although they have different crystalline size and morphology due to the ionic substitution (e.g., Na^+^, Mg^2+^, CO_3_^2−^) in BHA, both samples exhibited similar biological activity in terms of biocompatibility and non-toxicity in human osteoblast cell cultures.

Egg-shell structure, which has been frequently described in the literature, is mainly composed of calcite (94–95%) with trace amounts of inorganic components, such as magnesium carbonate or calcium phosphates [115]. The synthesis of HA from egg-shells is generally achieved by ball-milling and subsequent sintering procedures [115].

Aquatic bones and shells are reliable sources for the production of HA since they contain a high content of minerals, such as calcium carbonate in the form of calcite or aragonite, silica and calcium phosphate [22,60,61,62,63,64,116,117,118]. Two main routes are generally used to convert marine-origin material to HA: (*i*) hydrothermal synthesis and (*ii*) hydrothermal hot-pressing. Hydrothermal synthesis involves heating processes under alkaline conditions at a specific temperature and pressure, while hydrothermal hot-pressing involves supplementary a compacting process. The temperature used for calcination and alkaline treatment are crucial parameters influencing the crystallinity, grain size and specific surface area of the final product [22]. The optimal preparation temperature ranges between 200–250 °C. In contrast to the HA derived from bovine bones, HA prepared from aquatic sources is reported to be thermally stable at a temperature up to 1200 °C [22].

One of the advantage of non-synthetic HA routes in comparison to the synthetic HA fabrication methods is their lower cost [22,55]. In this sense, the production of HA from various wastes is an equally excellent and promising alternative [22]. For example, the use of egg-shell or sea-shell wastes, which represent the most abundant by-products of the food industry, has been found as a very promising sustainable resource to produce HA at a low cost with little impact to the environment [88,119,120,121]. Apart from the method of synthesis, the quality of non-synthetic HA (e.g., purity, grain size, properties) is strongly dependent on the biological source, location, age and fabrication process [1,55,92,93,94,99,100,104,109,111,112,116].

Another major advantage of this fabrication route of HA materials is the extremely low risk of immune reactions. In order to trigger an immune response, the antigen-presenting cells in the body need to find an organic compound that is identified as non-self, usually a protein/peptide or an oligosaccharide with a specific sequence. As previously mentioned, in the process of preparing animal-bone derived HAs, all organic compounds are completely destroyed by the high processing temperatures. The inorganic component of animal-bone HA is similar to the major mineral phase of human bone, thus being safe from immune responses. However, if one further dopes biological HA with low quantities of various cations (e.g., Cu, Co, Cr, Ni, Ag), rare allergic reactions could occur due to the fact that such ions act as haptenes [122] (as after binding normal proteins they modify their conformation forcing the immune system to act against them as non-self material).

### 2.2. Synthesis of Bulk Synthetic Substituted HA

Synthetic routes, even though costlier, offer the possibility to fine tune the properties of HA by cation and/or anion substitution, in order to boost its sustainability for specific applications (e.g., dental implants could be subjected to more acidic environment) and long-term performance.

The physico-chemical properties of substituted synthetic HAs (SHA) are extremely sensitive to the processing conditions and type of preparation method. The final features of synthesized HA (e.g., morphology, structure/crystallinity, composition, porosity, mechanical features and biological properties) will have a great influence on the overall in vivo performance of the bioceramic.

SHAs have been prepared by different methods, such as wet-chemistry methods (e.g., co-precipitation, hydrothermal, sol-gel), solid-state reaction, combustion, microwave and mechano-chemical synthesis [1,2,5,6,32,69,123,124,125,126]. The advantages and limitations of the HA fabrication methods, as well as their ability to produce bioceramics of different shapes and crystalline quality, were insightfully reviewed by Mucalo [91], Fihri [6], and Sadat-Shojai [127].

The most widely used synthesis methods are the wet-chemistry ones, with emphasis on the co-precipitation from the solutions of calcium, phosphate and selected dopant salts [1,5,6,126]. The co-precipitation method, working at temperatures ranging from room temperature to −200 °C, usually provides nanocrystalline HA, thereby with high specific surface area [126,128]. Nevertheless, it was demonstrated that the size and shape of HA powder particles, prepared by co-precipitation, can be controlled/tailored by reactants involved in synthesis, concentration of solutions, pH of the environment reaction, acid addition rate, reaction temperature, and/or post-synthesis thermal-treatment [2,6,127]. Generally, in order to obtain doped HA with good crystallinity, the addition of the reactants requires intense stirring, while the time of maturation of the precipitate should be longer. The crystal shapes can be controlled by the reaction temperature [2,6,127,129].

### 2.3. Fabrication of Substituted HA Coatings

The poor mechanical properties of HAs have limited their stand-alone use to non-extreme load biomechanical bearing applications [34,130,131]. The coating of metallic implants and endo-prostheses with HAs has provided the opportunity to combine the excellent mechanical performances of the substrate with the superior biological properties of HAs (with emphasis on its ability to form a rapid and strong interfacial bonding with the host bone). Consequently, the implant-type coatings represent nowadays one of the prominent clinical applications of HA, and the only current use in load-bearing implantable devices.

Bioactive HA coatings have been applied to both metallic [1,2,35] and thermoplastic polymers (e.g., PEEK—polyetheretherketone) [132] substrates. SHAs could effectively improve the biological performance of metallic implants, when applied as coatings, due to their superior properties provided by the controlled doping [20,35].

Nowadays, the commercial solution for producing HA implant coatings on titanium substrates is plasma spray. Although this implant design had certain clinical success [133,134,135,136], it is marred by a series of deficiencies which raise queries about its long-term operation: due to their typical high thickness (>50 μm) the implant coatings are susceptible to poor adherence and delamination, whilst the high-temperature process often induces residual phases with unpredictable degradation rates in the internal body media. Currently, a plethora of coating techniques exist as possible alternatives. HA coatings were fabricated by sol-gel [137,138], electrophoresis (EPD) [137,139,140], electro-chemical deposition (ECD) [141,142], ion-beam assisted deposition [132,143], micro-arc oxidation [144,145], and biomimetic deposition from supersaturated simulated body media solution [146,147,148]. Emerging synthesis approaches include physical vapour deposition techniques such as: magnetron sputtering [35,67,68,70,131,135,149,150,151], pulsed laser deposition (PLD) [35,89,97,98,105,135], pulsed electron deposition [35,135,152,153,154,155], and matrix-assisted pulsed laser evaporation [117,135,156,157,158,159]. The advantages and limitations of each technique have been reviewed insightfully by Narayanan and Bosco et al. [160,161] and Surmenev [131]. Both biological [89,97,98,153,154] and synthetic HAs [131] have been used as source materials for the fabrication of implant coatings.

**Figure 3:**
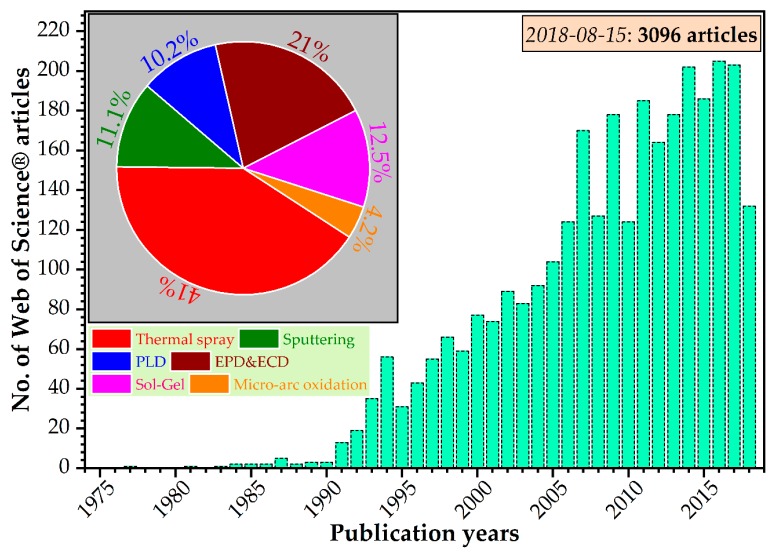
Yearly distribution of scientific articles published on the hydroxyapatite coatings topic over the 1975–2018 period (15th of August 2018). Database: Clarivate Analytics—Web of Science^®^ Core Collection. Coupled «title» and «topic» search keywords: “hydroxyapatite”, “hydroxylapatite”, “HA”, “HAp”, “Ca_10_(PO_4_)_6_(OH)_2_”, “Ca_5_(PO_4_)_3_(OH)”, “coating”, “film”, and “layer”. Inset: Frequency comparison of the most used technologies for coating fabrication.

Significant efforts have been devoted to the fabrication of reliable coatings, and from the total of published papers, this specific HA niche represents more than 14% (Figure 3) of the research works. When comparing the most used deposition techniques, it is evident that the thermal spray family (here including plasma spray, cold spray, detonation spray, flame spray, high-velocity oxy-fuel spray, high-velocity atmospheric spray, and high-velocity suspension flame spraying) was the prominent research choice, followed by the EPD and ECD methods (Figure 3—inset). All the mentioned deposition technique variants can be adopted for the fabrication of SHA coatings.

## 3. Cation-Substituted Hydroxyapatites

Besides the biomedical field usage, which will be comprehensively reviewed in the following, cation-substituted HAs have been envisaged to be employed in a variety of other type of applications (Table 2).

In the framework of this review the cation dopings were categorised with respect to the table of Elements nomenclature of “blocks”, thereby, based on their electronic configuration (i.e., the highest-energy electrons for each cation species in a block belong to the same type of atomic orbital).

### 3.1. s-Block Cation-Substituted Hydroxyapatites

Lithium (Li) is present in organisms as trace metal and it is also used as treatment in psychiatry (for bipolar disorder) or for haematological conditions (e.g., neutropenia, aplastic anaemia). Li compounds can be prescribed as prophylactic or adjuvant in the treatment of leukopenia or thrombocytopenia induced by chemotherapy [212,213]. Moreover, Li treatment is involved in haematopoiesis by enhancing the production of G-CSF (granulocyte colony stimulating factor) and by stimulating the proliferation of pluripotent stem cells [214]. Importantly, Li has also demonstrated a positive role in bone biology [215], boosting fracture healing by activating the canonical Wingless integrated (Wnt)/β-catenin signalling pathways that are important in the inflammatory phase of fracture repair [216,217,218,219,220]. Since the Wnt pathways are activated, the differentiation of mesenchymal progenitor cells into osteogenic agents will be successfully induced.

Li-HA showed a lower degradation rate than pure HA in simulated body fluid (SBF), whilst still inducing the in vitro formation of a biomimetic apatite layer [221]. Li-doped HA scaffolds showed efficient osteoblast proliferation and enhanced viability when tested in vitro and also revealed good osteogenesis and angiogenesis potential when studied in vivo (on Japanese white rabbits) [221,222]. By doping HA with Li^+^, the osseointegration is accelerated and the anchorage of bone metallic implants to host tissue is improved [89]. At the studied concentrations (≤2 at.% Li), Li-HA showed good biocompatibility, without traces of cytotoxicity [221,222,223]. The in vivo tests on animal model (rabbit) demonstrated the capability of Li-doped (1.5 at.%) HA scaffolds to induce the formation of new bone with well-defined trabeculae, as evidenced by histological detection of haematoxylin and eosin and Masson staining [224].

Sodium (Na) is a very important electrolyte in all the living organisms. In humans it has vital roles in transmission of nerve impulses, muscle functions, regulation of fluid balance, heart activity and in bone metabolism [225,226,227,228]. As a dopant in HA, Na^+^ enhances the biomineralization capacity (i.e., carbonated hydroxyapatite formation) in SBF [227,229] and increases the coating adhesion on reinforced carbon fibres [229]. Na-HA coatings are biocompatible (when tested on mouse skull osteoblastic cells, MC3T3-E1), increasing cell proliferation [229]. Microscopy investigations showed an enhanced osteoconduction of Na-HA with respect to pure HA, highlighted by the formation of a thick and dense new bone in the calvarial defects of rabbits 4 weeks after implantation [227].

Potassium (K) is known as a beneficial element for dental health since it influences the apatite nucleation and biomineralization processes [230]. Incorporating K^+^ in HA will positively affect the thermal stability [231]. K-doped HA is beneficial for protein adsorption and it could be used in needle-free trans-dermal delivery vehicles for proteins/antigens [232,233].

Magnesium (Mg), the fourth most abundant cation in the human body, has a high biocompatibility with living cells and an important role in bone health by stimulating osteoblast proliferation at the early stages of osteogenesis [234]. Deficiency of Mg causes bone loss. Mg-substituted (5.7 at.%) HA has a comparable composition, morphology and crystallinity to the biological apatite, without cytotoxic effects [5,235]. Mg doping might induce a partial decomposition of the HA into β-tricalcium phosphate in the temperature range 650–1000 °C [234]. The Mg doping effect on biological properties of HA was tested on a wide palette of compositions: 1–53 at.% Mg (with respect to [Mg/(Mg + Ca)]∙100]) [236,237,238,239,240]. Mg is also a prominent constituent of biodegradable metallic implants due to its biocompatibility and biodegradability in the physiological environment [2,241]. 

The formation of a biomimetic apatite-like layer was found to be stimulated in SBF solution by increasing the Mg doping concentration in HA from 1 to 3 at.% [242]. Mg-HA structures ensured endothelial and osteoblast (OBs) cells survival and spreading, improved OBs adhesion and promoted cell proliferation [236,240]. By contrast with most biocompatibility studies performed on several type of cell lines [236,239,240,243], Lima et al. found that Mg-HA materials induced apoptosis of human monocytes (isolated from blood) at doping concentrations as low as 1 at.% [238]. However, the high dissolution rate of Mg-HA still needs to be addressed, since it influences the cell viability and overall cytotoxicity [1,67]. Mg-HA showed bactericidal effects against Gram-positive (*Staphylococcus aureus*) and Gram-negative bacteria (*Pseudomonas aeruginosa* and *Escherichia coli*) at doping concentrations starting from ~6 at.% [237]. The in vivo tests on animal models (New Zealand White rabbits) indicated that Mg-doped (15 at.%) HA used as filling for femoral bone defects had enhanced osteoconductivity with respect to the commercial stoichiometric HA [235].

Strontium (Sr) is one of the most promising doping cations, since it is able to promote osteoblast cell proliferation and stem cell differentiation, therefore enabling new bone formation and fostering significant roles in osseointegration [243,244,245,246]. Being a trace element in natural bone (more abundant in new bones than in aged ones), Sr^2+^ is easily incorporated and accepted by tissues. Strontium ranelate (SrR) has been administered widely as a treatment for osteoporosis [247], due to its ability to boost osteoblasts proliferation and reduce osteoclast differentiation, allowing for accelerated bone healing even for elderly patients [215]. SrR influences NF-κB and Wnt/b-catenin signalling pathways in the mesenchymal stem cells, promoting the proliferation of osteoprogenitor cells [248,249]. SrR can also stimulate angiogenesis through the PI3K/AKT/mTOR signalling pathway [248].

Sr doping in HA was tested over a wide concentration range (1–40 at.%) [240,244,245,246,250]. The ability of Sr-HA to induce an enhanced formation of biomimetic apatite was demonstrated both in SBF [246,251,252] and cell culture medium (i.e., modified Eagle’s medium) [244]. Regardless of Sr content, no signs of cytotoxicity were reported, with Sr-HA unanimously promoting osteoblasts proliferation and differentiation, corroborated with mitigation of the osteoclasts activity. Furthermore, the in vitro biological effect of Sr-doped HA bone cements on mesenchymal stem cells (MSCs) and OBs suggested that the bioceramic was able to respond with great specificity to each type of cell [240,244,245,247,253]. Besides preventing bone resorption by reducing the osteoclasts activity [245], Sr doping improves the mechanical properties of HA [250,254,255,256]. The in vivo tests in animal model (JW rabbits) coupled with optical microscopy (following hematoxylin and eosin and Masson staining) and micro-computed tomography studies, showed that higher volumes of new bone are formed in the case of Sr-HA-based scaffolds with respect to pure HA ones [257]. Similarly promising results were obtained also when performing in vivo tests on rats, with Sr-HA being able to reduce the area of calvarial bone defects and induce the formation of a denser bone tissue with respect to the pure HA groups [249].

Barium (Ba) has been used in dental cements as filling agents in root canals, due to its excellent mechanical properties and low cytotoxicity [258,259,260]. But, to date, there is only one paper that studied Ba-doped HA (mono-doping) for biological applications [261]. In this study, the Ba-HA antimicrobial activity was tested against *S. aureus, Bacillus megaterium DMS 32, E. coli, Klebsiella pneumonia, and Candida albicans*, but no relevant efficacy against these pathogen colonies was noticed [261]. The in vitro bioactivity tests performed in SBF on pure and Ba-doped (4 and 10 at.%) bi-phasic calcium phosphates (91 wt.% HA + 9 wt.% β-tricalcium phosphate), disclosed an increased formation of biomimetic apatite with the Ba concentration [262].

### 3.2. p-Block Cation-Substituted Hydroxyapatites

There are only a few p-block elements that were tested as cation dopant in HA: aluminium, gallium, indium, bismuth, and tellurium.

Aluminium (Al) doping in HA had been proposed for its potential use in biomedicine, due to good biocompatibility in mouse fibroblasts cell cultures. It was highlighted that the cell viability decreases gradually with the increasing Al^3+^ concentration (tested in the range 0.5–2.5 at.%) and incubation time. Al-HA is biocompatible when added in an amount up to 1 mg mL^−1^ [263]. 

Gallium (Ga) seems to not substitute Ca^2+^, but it enters on interstitial positions or it is adsorbed/chemisorbed on the particles surface [264,265]. Regardless of its site occupancy in the HA lattice or surface, Ga^3+^ is a promising candidate for biomedical applications due to its demonstrated Ga-HA biocompatibility (in RAW264.7 cell cultures) and inhibitory antibacterial effect on *P. aeruginosa* [264,266].

Indium (In) doping of HA improved the osteoblasts activity by increasing their adhesion and differentiation rates [267].

Bismuth (Bi) doped HA was found to be cytocompatible with human osteoblasts [267], but induced certain levels apoptosis of human blood monocyte [238]. Bi^3+^ is normally not found in the human body, but when doping HA with Bi the adherence and differentiation of OBs could be enhanced. Bi-HA possesses the ability to induce the formation well-developed bone-like apatite layers after 1 month of immersion in SBF [268]. Bi^3+^ doping increased the dissolution rate of HA and elicited an antibacterial effect against *S. aureus* and *E. coli*, which makes Bi-HA a pertinent candidate for bone implant applications [267,269]. Bi-HA (scaffold)—polyurethane (matrix) composites were tested both in vitro (human osteoblast-like cells, MG63) and in vivo (in rabbits—subcutaneous and in intraosseous tibia sites) [268]. Excellent mechanical properties, antimicrobial activity against various pathogens, high osteoconductivity and in vitro biocompatibility was revealed. The in vivo investigations demonstrated the osteogenic potential of Bi-HA—polyurethane composite, with the authors advocating for a proper biomimetic microenvironment for bone regeneration with excellent cytocompatibility [268].

Tellurium (Te) is a metalloid element that has antioxidant and pathogen-inhibiting features [270,271]. The use of Te as a low-level doping element in HA promoted antimicrobial activity against Gram-positive (*S. aureus*, *Bacillus subtilis*, *Micrococcus sp.*) and Gram-negative (*P. aeruginosa*, *Klebseilla sp.*, *Proteus mirabilis*, *Shigella dysenteriae*) bacteria and fungi (*Candida albicans*) [272]. However, more insightful biocompatibility studies should be performed for Te-HA materials, prior to drawing a safe conclusion on their potential for biomedical applications.

### 3.3. d-Block Cation-Substituted Hydroxyapatites

Silver (Ag) is known to be a highly effective inhibitory or antimicrobial agent for Gram-positive and Gram-negative bacteria, as well as for fungi [250,261,273,274,275,276,277,278,279,280,281,282,283]. This dopant is preferred for HA applications in dentistry and orthopaedics, where the hazard of implant infections has a high rate. In this respect, the long-term release of Ag^+^ ions [284], could be an optimal solution. The main issue that needs to be carefully addressed is the cytotoxicity of Ag^+^, in order to determine the trade-off concentration values that will be both effective against microbes and safe for host tissues. Several studies investigated the toxic effect of Ag^+^ on pathogens and on various cells culture lines [250,273,280,281,282,283]. Although there is some dispersion in the tested compositions of Ag-HA (0.5–5 at.%), it seems that the optimum silver doping is situated below the 2 at.% threshold [250,273,280,281,282,283]. The fabrication or preparation of Ag-doped HA have also an influence on its biological activity. Ag-HA coatings with a Ag content of ~1.7 at.%, synthesized by plasma spray, had highly effective bactericidal properties against *P. aeruginosa*, but also a slight cytotoxic effect on human osteoblast hFOB 1.19 cell line, with cells showing premature apoptosis, delayed differentiation or even death [250]. In the case of processed powder forms of Ag-HA, the biocompatibility with human osteoblast cells was not affected by Ag content (at concentrations up to 1.5 at.%), while maintaining a bacteriostatic effect [280]. Although the antimicrobial properties of Ag-HA were thoroughly investigated, the cytotoxicity coupled with the ion release rates has not been too frequently addressed [274,275,276,277,278,279,281]. The antibacterial spectrum of Ag-HA is very wide, but still there are some pathogens that are not affected by Ag^+^, such as *B. subtilis, Enterococcus faecalis* (ATCC 29212) [275] and *Serratia marcescens* (0804) [276]. In vivo evaluation of Ag-HA efficiency against Methicillin-resistant *S. aureus* (MRSA) was performed on Sprague-Dawley rats [283]. The Ag-HA implants reduced the MRSA biofilm formation, without inducing argyria (or any other kind of skin disorder) or being harmful to brain, kidney, liver or spleen. Furthermore, a good biomineralization capacity was disclosed for Ag-doped (0.13–5 at.%) HA by in vitro assays performed in SBF [285] and McCoy culture medium [286].

Zinc (Zn), besides being one the most abundant trace cation of bones, plays a crucial part in several body functions, markedly being a cofactor in hundreds of enzymes involved in bone functions and metabolism. Doping HA with Zn^2+^ increased the osteoblast cells viability, adhesion, spreading, proliferation and differentiation, and stimulated osteogenic activity, bone in-growth and healing [243,267,287,288,289]. Restoration of normal Zn^2+^ and citrate levels have been observed to improve the bone quality in age-related osteopenia. High osterix levels (induced by the activation of runt-related transcription factor 2-Runx2) determines the increase of ZIP1 transporter activity, thus elevating the intracellular Zn levels [290]. Furthermore, the high Zn levels have been linked to the high concentrations of citrate in the extracellular matrix, leading to a rapid formation of HA and citrate incorporation into HA [290]. In some situations, the incorporation of Zn in HA powders had a toxic influence on cells (i.e., HepG2 cells—human hepatocytes [65,291]), as a consequence of Zn-HA particle sedimentation over cells. Excellent bioactivity of Zn-doped (2.4 at.%) HA was evidenced after only 3 days of soaking in SBF solution [292]. Zn-doped HA was confirmed as an effective antimicrobial agent against Gram-positive and Gram-negative bacteria frequently occurring at the implant site: e.g., *S. aureus*, *Streptococcus mutans*, *Staphylococcus epidermidis*, *Enterobacter aerogenes*, *E. coli* [65,274,288,289,291,293,294,295]. The Zn^2+^ release acted against fungal infection, the 72 h *C. albicans* biofilms being strongly reduced at Zn concentration of 3 at.% [277]. However, in dark conditions, at a lower Zn content (i.e., 1 at.%) the number of *C. albicans* cells was also noticeably decreased [296]. Zn-HA was proficient in the case of cold-light bleaching-treated enamel remineralisation [289]. Zn^2+^ doping had a positive effect on the inhibition of bacterial plaque formation on enamel and on the improvement of the enamel remineralisation in dental prosthetic restoration. However, at high Zn concentrations (≥2 at.%) the biocompatibility was affected, even though Zn-HA was efficient against enamel bacteria growth (*S. mutans*, *Lactobacillaceae*, and *Streptococcus sobrinus*), whereas 1 at.% of Zn doping enhanced both osteoblast proliferation and antibacterial properties. The vast majority of Zn-doped HA is confined in the 0.1–4 at.% [Zn/(Zn + Ca)∙100] doping range [66,243,274,277,281,287,291,293,296]. However, the influence of higher zinc content (up to 50 at.% as [Zn/(Zn + Ca)∙100]) on the biological activity of HA was also reported [288,294]. The best results in terms of biocompatibility, osteoconductivity and antimicrobial activity seem to be achieved for Zn^2+^ concentrations of ~1–2 at.% [243,274,282,288,289]. Remarkably, the in vivo tests on animal model showed the Zn doping capability to enhance the new bone formation in comparison to pure HA, when implanted in rats [297] and rabbits [298], for one and two months, respectively.

Copper (Cu) is an important micronutrient in organisms, being involved in the metabolic processes and in the proper functioning of organs. Cu-doped HA is an acknowledged antimicrobial agent, acting against both Gram-positive (*S. aureus*) and Gram-negative (*E. coli*) bacteria, as well as fungi (*C. albicans*) [299,300]. The antibacterial activity of Cu-HA powders seems to be highly dependent on the doping concentration: low Cu^2+^ content (<0.5 at.%) was efficient in combating the Gram-negative bacteria [300,301], while the Gram-positive microorganisms are sensitive only to higher Cu doping level (~2 at.%) [299]. The antimycotic effect is revealed from low to high Cu^2+^ concentration ~0.4–5 at.% [299,300]. Cu^2+^ ions released from Cu-doped (~2.4 at.%) HA coatings have a strong bactericidal effect against *E. coli* colonies (bacteria cells were reduced by more than 75%) [302]. In addition to antimicrobial behaviour, the doping of HA with Cu might be beneficial for inducing protein adsorption, osteogenic differentiation, bone-like apatite nucleation and growth at the implant site [302,303]. For instance, the superior bioactivity of Cu-doped (2.4 at.%) HA with respect to the pure compound was demonstrated by Huang et al. [302] after 10 days of immersion in SBF. Moreover, HA coating doped with low Cu^2+^ contents (~2 at.%) exhibited good cytocompatibility toward mouse skull osteoblasts (MC3T3-E1). An IP6-assisted hydrothermal method was used to fabricate Cu-HA nanoparticles, with a theoretical Cu concentration of ~5 at.%, that were effective against *S. aureus* and *E. coli* stains, while being cytocompatible in a rat calvaria osteoblast (RCO) cell line and promoting osteogenic differentiation [303]. However, contradictory cytotoxicity results were also published in the case of Cu-doped HA. If the previous two presented cases indicated a good biocompatibility at doping levels of 2–5 at.% [302,303], other researchers reported on the alarming cytotoxicity of 1 at.% doped Cu-HA on to Balb/c 3T3 clone A3 mouse fibroblasts and on human foetal osteoblasts (hFOB 1.19) cell lines [238,301]. Moreover, Lima et al. [238] revealed a significant level of apoptosis when interacting with human monocytes (isolated from blood). Altogether, it is suggested that besides doping level, the synthesis technology as well as the testing cell line play prominent roles on the biological performance of Cu doped-HA.

Manganese (Mn) is a trace ion in organism, being involved in several metabolic processes. As a doping element in HA, Mn can increase the bonding strength between HA film and metallic (Ti) implant substrate, while enhancing the corrosion resistance [194]. The Mn-HA possesses the ability to induce the nucleation and growth of biomimetic apatite layers in SBF [304,305,306]. Mn-HA stimulated cell viability and osteoblast proliferation, enhanced protein adsorption on the coating surface, thereby, and overall improved the metallic implant biocompatibility [305,307,308]. Mn-HA showed no cytotoxicity in the studies performed by Huang et al. [305], Li et al. [308], and Zilm et al. [309].

Iron (Fe) takes part in various organism functions, including bone metabolism. Fe promotes apatite nucleation (as demonstrated by both in vitro assays in SBF [310] and in vivo tests in sheep models [311]), enhances osteoblast adhesion, division and proliferation, and induces osteogenic function [308,309,312]. Moreover, Fe-HA exhibited antimicrobial effects on *S. aureus* and *E. coli*, while being biocompatible with human osteosarcoma cells (SaOS2), and increasing cell viability without any signs of cytotoxicity [312]. Magnetic Fe-HA nanoparticles damaged HepG2 cancer cells through hyperthermia processes [313,314]. A significant and fast effect on murine colon cancer was achieved within two weeks of Fe-HA action [313]. Such materials showed good biocompatibility and little toxicity when injected subcutaneously [313]. However Lima et al., previously mentioned in the case of Mg-, Bi- and Cu-doped HAs, have shown that the 1 at.% doped Fe-HA, induces, as in the case of the other dopants, a significant level of human monocyte cell apoptosis [238].

Titanium (Ti) is an excellent choice for biomedical applications mostly due to its biocompatibility (related to its surface passivation that produces a thin hermetic TiO_2_ layer) and high mechanical strength [315]. Besides its use for the fabrication of medical devices (in either pure or alloyed form), Ti^4+^ can be integrated as substitutional dopant in HA to enhance cells viability, proliferation and differentiation, along with the stimulation of the extra-cellular matrix mineralization [68,316]. The in vitro formation of biomimetic apatite layers on top of Ti-HA was evidenced by SBF testing irrespective of doping level (no pure HA control data were provided) [317,318]. The biological activity of Ti-HA coatings is dependent on the Ti substrate surface: a rough metallic substrate enhanced the production and mineralization of the bone matrix compared to a smoother one, therefore enhancing the osseointegration capability [316]. Ti-HA showed slight bactericidal effect against *E. coli*, especially when the system is UV irradiated [177]. An effective antibacterial capability is achieved at ~13 at.% Ti, although this effect is accompanied also by a slightly cytotoxicity on human foetal osteoblast cells [301]. Doping HA with Ti was proved to be beneficial for the increase of protein adsorption [177] and improvement of the mechanical properties (i.e., bonding strength, hardness and elastic modulus [68,105]).

Chromium (Cr) is a trace element in the human body that is essential to metabolize sugars and fats. Doping HA with Cr might be beneficial for biomedical applications, but the cytotoxicity of such materials needs to be properly assessed. In vitro studies performed on cervical cancer cells (HeLa) and mouse fibroblast cells indicated that in both cases Cr-HA nanoparticles are cytocompatible up to a concentration of 800 μg mL^−1^ and for a duration of 24–48 h [319]. In terms of haemocompatibility, the use of a low content of Cr dopant (0.5 at.%) is compulsory, as the blood cells are highly susceptible to cytotoxic stimuli [319]. Tests performed on *Drosophila melanogaster* Meigen genes have shown that Cr-doped (1 at.%) HA powders do not exhibit genotoxicity [320].

Cobalt (Co) is an important element in human body, being a constituent of vitamin B12. Co ion doping of HA enabled antimicrobial activity against *S. aureus*, *Microcosus luteus*, and *Shigella flexneri*, but no such effect was encountered against *P. aeruginosa* [321]. For a low doping level (i.e., 0.37 at.%), Co-HA sustained the human osteosarcoma cell viability, proliferation and differentiation, endorsing both osteogenic and proangiogenic properties [322]. A case of cobalt-doped HA (1 at.%) apoptosis on human blood monocytes was reported, despite the fact that it simultaneously enhanced the osteoblasts adhesion [238]. The in vivo tests on animal models (white female Wistar rats) indicated that Co-HA stimulates the osteogenesis inside mandibular defect, 6 months after implantation [323].

Tantalum (Ta) doping of HA was rarely explored [324,325]. Ta-HA was shown to increase human osteoblast cell proliferation, hinder charge storage ability, but at the same time induce the partial decomposition of HA into β- and α-tricalcium phosphate [324].

Nickel (Ni) was shown to possess a dose-dependent cytotoxicity, and was tentatively advanced for biomedical applications [326,327]. When incorporated in HA, Ni had a positive effect on human osteosarcoma MG63 cell viability, proliferation and differentiation, with the adhered cells embedded into the bone matrix. Specifically, Ni-HA with low doping concentration (≤2.4 at.% Ni) is osteoconductive and proangiogenic [328]. Moreover, when used as a substitution co-dopant (together with Mg^2+^ and (SiO_4_)^4−^), Ni^2+^ enhanced the antibacterial effect against *E. coli* and *P. aeruginosa* [329].

Molybdenum (Mo) can be used as well as a doping cation in HA. The only paper found in this regard emphasized that Mo-HA nanorods can be used as antimicrobial agents in bone cement engineering, due to their bactericidal (*S. epidermidis* and *E. coli*) and anti-fungal (*C. albicans*) properties [330]. However, prior to being further recommended as a bone cement component, Mo-HA cytocompatibility should be assessed as well.

Yttrium (Y) is a d-block element that improves HA biocompatibility in human osteoblasts cell cultures [331,332]. Moreover, Y-HA was used as therapeutic agent for radioactive synovectomy in haemophilic synovitis [333].

Cadmium (Cd), although renowned for its toxicity, was tested as doping cation in HA to explore its action mechanisms. As the Cd content increased, the levels of DNA damage were substantially augmented in the liver of zebra fish, eventually causing death [334]. Cd-HA had also a detrimental effect on the growth of plants [335].

Tungsten (W) doped HA has great catalytic activity. W-HA enhanced the biosorption and adsorption of methyl orange by *E. faecalis*, having massive decolourization as a consequence [336]. This W-HA feature can be used in wastewater decontamination.

Hafnium (Hf) shows a great potential for oncological applications. Its high electron density and photo-luminescent properties make it a good candidate for photodynamic therapy [337,338]. In this respect, Hf-doped HA was tested in vitro on A549 human adenocarcinoma and in vivo in mice with lung cancer [339]. In vivo studies showed that when Hf-HA nanoparticles (NPs) are bombarded with ionizing radiation, the mice tumour growth was inhibited due to cell apoptosis. In vitro studies revealed also a high cytotoxicity towards human adenocarcinoma due to the formation of reactive oxygen species, while Hf-HA NPs interacted with ionizing radiation [339].

### 3.4. f-Block Cation-Substituted Hydroxyapatites

Recently, rare-earth metals (REM) (e.g., lanthanides and scandium) and actinides have attracted great interest in the orthopaedic field due to their high biological activity and ability to replace calcium ions in HAs [1,126,340]. Different lanthanide and actinide doping in HA have been attempted, such as lanthanum (La^3+^) [267,340,341,342,343], cerium (Ce^3+^) [344,345,346,347,348,349], praseodymium (Pr^3+^) [345,350,351], neodymium (Nd^3+^) [128,345,352,353], samarium (Sm^3+^) [354,355,356,357,358,359], europium (Eu^3+^) [353,360,361,362,363,364,365,366,367], gadolinium (Gd^3+^) [128,354,368], terbium (Tb^3+^) [345,353,361,369,370], holmium (Ho^3+^) [371], erbium (Er^3+^) [353,372], thulium (Tm^3+^) [360], ytterbium (Y^3+^) [373], and uranium (U^3+^) [374,375].

The incorporation of lanthanides and actinides ions into HA is of significant interest for biomedical applications due to their excellent affinity for Ca^2+^ sites. In particular, for trivalent lanthanides this strong affinity is explained by an ion-exchange mechanism; the binding constant for the exchange increases as the ion size decreases [376]. When trivalent cations substitute Ca^2+^ ions, the charge imbalance is compensated for by either the generation of vacant cation sites or a loss of a proton from (OH)^−^, and the ion-exchange ability depends strongly on the fluctuation of charge density induced by the adaptation of the lattice parameters [50,376]. The charge density increases as the lanthanides ion size decreases [376]. The lanthanides are well-suited elements for cationic substitution due to their similarities in ionic radii with Ca^2+^, donor atom requirements and coordination polyhedron geometries [376]. After substitution with REMs, a general decrease of crystallinity and increase of surface area was observed for doped HAs with respect to the pure phase [5,377]. REM or actinides-HA composites exhibited exceptional luminescence properties and are promising for application in biological fluorescence labelling (e.g., magnetic resonance imaging, multi-imaging diagnosis on single photon emission computed tomography (SPECT)). Their fluorescence is characterized by narrow emission bandwidths, high photochemical stability and long fluorescence lifetimes [1,126]. However, the exploration of the biological response of such doped HA materials is still in its infancy. In particular, the cytotoxicity effects are still open questions. The authors of this review advocate for the necessity of clarifications in this respect.

Lanthanum (La) doping can improve various physico-chemical properties of HA such as the thermal stability, resistance in acidic and physiological media, or inhibition of bone resorption [340,378]. Mechanical properties, such as tensile strength or micro-hardness, have been found to be enhanced with the increase of La^3+^ content in HA [340,379]. Also the addition of La ions improved the cell response and the antibacterial efficiency [378]. Joshy et al. observed in La-doped HA, prepared by the sol-gel technique, an antibacterial activity against Gram-positive (*S. aureus* and *Bacillus*) and Gram-negative (*E. coli* and *Pseudomonas*) [379]. Lou et al. prepared by a wet-chemical method La-doped HA with a doping atomic concentration up to 30%, and further used it to fabricate implant coatings on Ti substrates by dip-coating [340]. No phase decomposition was observed. Their results indicated good bonding strength at the coating-substrate interface, accompanied by good angiogenesis and cytocompatibility in mouse calvaria MC3T3-E1 cell cultures (for La concentrations below 20 at.%) [340]. Excellent biocompatibility was also observed in La-doped HA powder with similar doping range for a mouse L929 fibroblast cell line [342]. However, Jadalannagari et al. reported a cytotoxicity of 40% for adenocarcinoma MCF-7 cell cultures exposed for 72 h to La-doped (10 at.%) HA at doses in the range of 5–100 µg/mL [380]. For similar La-HA powder/cell media ratios, no cytotoxicity against human embryonic kidney (HEK) and MCF-7 cell lines was observed in the case of La-doped (2 at.%) HA. The viability was ~87% after 72 h of cell culturing [380]. Thereby, promising prospects of La-HA use in biomedical applications could emerge in the near future.

Cerium (Ce) cation is characterized by an ionic radius and electronegativity similar to Ca^2+^. Ce can easily substitute calcium, accumulate in small quantities in bones, and boost the bone metabolism and the biomimetic HA-forming ability [1,344,381]. Moreover, Ce participates in the prevention of dental cavities, reduction of enamel demineralisation, acts as an antioxidant, and provides high thermal-phase stability [344,381,382]. In vitro biomimetic apatite formation, after being soaking in SBF for periods of 2–3 weeks has been noticed for Ce-HA materials [383,384]. Ce can also stimulate the antimicrobial activity, pathogen inhibition and regenerative properties [344,377,381,382]. Both Ce^4+^ and Ce^3+^ cations are present in Ce-substituted HAs, but in proportions dependent on experimental procedure [348,349,385]. Various studies have reported the antibacterial properties of Ce-doped HA for Gram-positive (e.g., *S. aureus* [344,348,377,381,382,385], *Lactobacillus* [377], and *B. subtilis* [348]) and Gram-negative (e.g., *E. coli* [348,377,381,382,385]) strains. However, discrepancies exist in the reported Ce content able to induce an antibacterial effect. For example, Lin et al. synthesized by the sol-gel supercritical fluid drying method Ce-HA materials with a [Ce/(Ce+Ca)·100] atomic proportion ranging from 0 to 20 at.%. They obtained improved antibacterial properties against *E. coli*, *S. aureus*, and *Lactobacillus* when adding Ce-doped (>8 at.%) HA NPs in concentrations of 100 mg/mL Ce-HA nanoparticles [377]. However, no indications of these materials’ cytocompatibility were provided. The antibacterial activity for such high Ce doping levels was also confirmed by Sundarabharathi et al. [344] against *P. aeruginosa* and *S. aureus*, for sol-gel prepared Ce-doped (10 at.%) HA, and G. Ciobanu et al. [385], against *E. coli* and *S. aureus*, for co-precipitation synthesized Ce-doped (10, 20 and 25 at.%) HA. However, some studies report antibacterial activity below 8 at.% Ce doping levels, specifically in the range for 0.3–1.25 at.%, for both co-precipitation [382] and sol-gel [348,381] Ce-doped HAs. Since Gram-negative bacteria have a less complex and thinner cell wall, it is expected that they are more sensitive to antibiotics than Gram-positive bacteria [348]. Although most studies agree on an enhanced inhibition zone for Gram-negative bacteria than for Gram-positive ones [348,381,382,385], discrepancies have been observed by Lin et al. [377] for *S. aureus* vs. *E. coli*, Sundarabharathi et al. [344] for *S. aureus* vs. *P. aeruginosa*, and Priyadarshini et al. for *S. aureus* vs. *E. coli* and *P. aeruginosa* or *B. subtilis* vs. *E. coli* and *P. aeruginosa* [348]. The antibacterial effect was found to increase with Ce content, if adding also Fe_3_O_4_ nanoparticles [381] or Sr ions [344].

On the basis of these studies it is possible to conclude that Ce induces significant antibacterial properties to HA for a wide concentration range (i.e., 1.25–25 at.%), without the formation of secondary/residual phases. The mechanism of interaction between bacteria and Ce-HA colloidal solution was described by the release of Ce cations which penetrate the negatively charged surface of the microorganism cell membrane, inhibiting the DNA replication [348]. Concerning the biocompatibility, high concentrations of Ce^3+^ cations are cytotoxic [344]. MTT assays supported by inverted microscopy images indicated a good biocompatibility in human osteosarcoma MG-63 osteoblast cells exposed to concentrations of Ce-doped (1.25 at.%) HA-NPs situated between 200–600 µg/mL, after 24 and 48 h incubation [348]. Cytotoxicity was encountered only for Ce-HA-NPs concentrations in the range of 800–1000 µg mL^−1^ [348]. Also, although a slight decrease in cell viability was observed in comparison to the control, the CCK-8 assay indicated no significant cytotoxicity against mouse L929 fibroblast cells, when a Ce-doped (5 at.%) HA powder prepared by co-precipitation was added to the medium at doses lower than 100 µg mL^−1^ [386]. Slight cytotoxicity was observed for doses of 200 and 500 µg mL^−1^ [386]. In contrast, in vitro cytotoxicity assessed by MTT against human lung A549 cells exposed to Ce-doped (10 at.%) HA-NPs concentrations of 100 µg mL^−1^ showed a significant decrease in cell viability [344]. Interestingly, Ce-doped (9 at.%) HA coatings, deposited on titanium substrates by DC pulse micro-arc oxidation, exhibited a good biocompatibility in mouse skull MC3T3-E1 cell cultures at 48 h [346]. It is worth noting that the cytotoxicity of Ce could be alleviated by adding Sr as co-dopant [344,387]. Biocompatible Ce-HA could also find applications as a fluorescent probe for cellular imaging or as an antioxidant agent.

Praseodymium (Pr) doping in HA was shown to be suitable for applications in radiotherapy [350,351].

Samarium (Sm) is another important REM element for biomedical applications, being a good candidate for cancer radiation therapies and bone pain treatment [1,357]. Furthermore, Sm is able to change the permeability of cell membranes and can be used in the treatment of synovitis [357]. Ciobanu et al. reported the synthesis of Sm-doped (0.2–0.5 at.%) HA powder by co-precipitation and studied the influence of Sm concentration on the antibacterial activity against Gram-positive (*E. faecalis* and *S. aureus*) and Gram-negative (*P. aeruginosa* and *E. coli*) strains. The antimicrobial activity for Gram-positive and Gram-negative bacteria was obtained at Sm contents of 0.2 and 0.5 at.%, respectively, for Sm-HA-NPs doses in the range of 0.125–1 mg mL^−1^ [355]. An antifungal effect against *C. albicans ATCC 10231* strain was also observed by colony-forming unit count (CFU) assay and confocal laser scanning microscopy (CLSM) images of live/dead fungus [357]. Sm-HA exhibited an excellent biocompatibility (in terms of cell viability and proliferation) in human foetal osteoblast cell (HFOB 1.19) cultures for doping levels up to at 5 at.% [355]. The results revealed that the Sm-HA powder is a good candidate to treat wounds and prosthetic joint infection. The enhancement of the osteoblastic performance, cell viability and antibacterial activity was also demonstrated by Sathishkumar et al. [354], when Sm was present in HA as co-doping along with Gd.

Europium (Eu), like Ce, is present in the human body, in small amounts, in the bones and liver [1]. Eu is an interesting element for the treatment of osteoporosis [365] and for promoting bone remodelling cycle [366]. Eu is easily incorporated in the HA crystal lattice due to their similar ionic radius. Eu-HA induces the in vitro formation of bone-like apatite in SBF [388]. Eu-doped HA (0.1–2 at.%) showed good antibacterial activity against Gram-positive *E. faecalis* and Gram-negative *P. aeruginosa*, at powder doses of 31–1000 µg mL^−1^ and 125–1000 µg mL^−1^, respectively [362,389]. The antibacterial action against Gram-positive *S. aureus* strain has been demonstrated at powder doses of 31–1000 µg mL^−1^ [389]. No antibacterial activity was found for Gram-negative *E. coli* even at low concentration of Eu^3+^. Furthermore, at 2 at.% of Eu, Iconaru et al. [389] observed a good fungicidal activity against *C. albicans*. Various studies have shown the excellent biocompatibility of Eu-doped HA. Frumosu et al. [363] synthesized Eu-doped (0.5 and 1.5 at.%) HA by co-precipitation and observed for up to 4 days the cell proliferation of osteosarcoma MG-63 cells. Ca_10-x_Eu_x_(PO_4_)_6_(OH)_2_ bioceramics (with x = 0.01 − 0.2) prepared by co-precipitation enabled the excellent proliferation of human embryonic kidney (HEK 293) cells, with no sign of cytotoxicity after 24 and 48 h [365,390]. Eu-doped (5 at.%) HA NPs, used in doses of 0.3–30 µg/mL was not found compatible with transformed human umbilical vein endothelial cells (T-HUVEC) [391]. In vitro tests with L929 mouse fibroblasts and ex ovo tests using aqueous injection into vitelline vein of chicken egg, were performed by Tesch et al. [392] for Eu-doped (10 at.%) HA at doses of 25–500 µg mL^−1^ and 500 µg mL^−1^, respectively. Their results indicated a cell viability of more than 80% after 24 h incubation and no toxicity (i.e., thrombosis and vascular lysis) [392]. Zheng et al. [393] indicated that Eu-doped (15 at.%) HA nanorods, prepared by the hydrothermal route, have excellent biocompatibility with pulmonary adenocarcinoma A549 and HeLa cells (i.e., viability of more than 100% after being exposed for 24 h at Eu-HA doses of 20–320 µg mL^−1^). Miranda-Melendez et al. [364] showed a low or absent cytotoxicity at 24 h, for Eu-HA materials synthesized by wet-chemical precipitation, when cultivating human gingival fibroblast (HGF-1) cell cultures with doses of 500–2000 µg mL^−1^ of HA having Eu doping contents up to 20 at.%. After 48 h incubation, the best Eu-HA biological performance was met for the 5 at.% doping. Amazingly, a low toxicity (i.e., HeLa cell viability of more than to 80%) was revealed for HA NPs with large contents of Eu (~9–17 at.%) used at high doses (10,000–30,000 µg mL^−1^) [366]. However, when coupled with 5 fluorouracil (5FU), a drug used for cancer treatment, Eu-HA shown the potential to kill HeLa cells, indicating the applicability of such composites as theranostic agents [364,366]. 

Terbium (Tb) has attracted extensive attention due to its multiple potential application in biomedical field when combined with HA, Tb being one of the most luminescent rare-earth biological probes due to its excellent emission feature with a main signal at 544 nm [209,394,395,396,397]. Furthermore, Tb exhibits excellent photocatalytic [394] and gene delivery [370] properties, as well as potential bactericidal activity and an ability to inhibit cancer cell development [398]. However, Tb is harmful for the human body at high concentrations [398]. CLSM images indicated that Tb-doped (2 at.%) HA samples, prepared by co-precipitation, showed a good biocompatibility with transformed T-HUVEC [361]. Wei et al. [399] synthesized Tb-doped (~17 at.%) HA nanorods by hydrothermal method, and showed via optical density analysis using Counting Kit-8 assay, an excellent MC3T3-E1 cells viability when exposed to Tb-HA concentrations of 25–100 µg mL^−1^ over a period of 7 days. The results were further confirmed by the unaltered morphology of the luminescent cells. The lack of in vivo toxicity in animal model of Tb-HA nanorods was also emphasized by the histological analysis of various organs of rats [399]. Zheng et al. [393] indicated that Tb-doped (15 at.%) HA nanorods, prepared by the hydrothermal route, have good biocompatibility with pulmonary adenocarcinoma A549 cells after being exposed for 24 h to colloidal concentrations between 20–320 µg mL^−1^. Their results have been also confirmed by CLSM analysis.

Gadolinium (Gd), dysprosium (Dy), and neodymium (Nd)-substituted HA composites have been widely used for such a purpose in magnetic resonance imaging (MRI) [392,400]. It is worth mentioning also that paramagnetic elements play an important role in multimodal imaging as contrast agents [392]. Gd^3+^ and Nd^3+^ have been also used as theranostic NPs for early stage diagnosis of cancer by near-infrared fluorescence techniques [352,368]. The thermo-luminescence properties of Gd-HA could also be used in gamma radiation dosimetry applications [401]. However, few studies concerning their biological activity were reported to date. 

Li et al. [128] synthesized nanocrystalline Gd-HA and Nd-HA with different doping levels (1, 4.8, 9, and 17 at.%) and observed a de-hydroxylation of HA without significant changes in the lattice parameters. The doped-HA samples showed a significant increase of the electrical conductivity in comparison to pure HA which is important for the electromagnetic sector and for the acceleration of bone fracture healing [128]. The agar diffusion method and live/dead cell assays indicated that all but one Gd-HA samples (i.e., 17 at.% Gd-doped HA) studied by Li et al. [128] were not cytotoxic for human foetal osteoblast (hFOB 1.19) cells at 24 h; 9 at.% Gd was the optimum concentration showing good biocompatibility. Li et al. [128] mentioned that the presence of non-coordinated or free Gd^3+^ cations could explain the toxicity of the 17 at.% Gd-doped HA, but more insightful biocompatibility tests are required, since the deleterious role of secondary Gd_2_O_3_ phase and possible Gd(OH)_3_ moisture cannot be excluded. Laranjeira et al. [402] synthesized Gd-doped (2.5–9 at.%) HA by the co-precipitation method and showed no in vitro cytotoxicity effect on human dermal microvascular endothelial cells (hDMECs) at any of the Gd doping concentrations. The morphology of the cells was not affected. Furthermore, the Gd-HA samples dosed to a concentration up to 4000 µg mL^−1^ were haemocompatible, non-haemolytic and non-thrombogenic, which is a crucial for magnetic resonance imaging (MRI) applications. Important to note, Laranjeira et al. [402] reported no phase separation or the advent of metallic oxides/Gd(OH)_3_.

Victor et al. [352] studied the biocompatibility at 24 h of the Nd-doped (at.% 11) HA NPs on L929 fibroblast cell line, by MTT and Live/dead cell assays, using doses of 10,000 and 20,000 µg mL^−1^. A cell viability of more than 90%, with negligible influence on their proliferation, was observed [352]. The increased uptake by HeLa cells of Nd-HA containing alginic acid-4-acetyl salicylic acid nano-platforms, from 4 to 16 h, was confirmed by Raman microscopic imaging, which indicated a growing cluster size and localization in the cytoplasm. Victor et al. [352] showed also that Nd-doped HA are able to deliver with great specificity anticancer drugs and simultaneously allow for fluorescence imaging, which would be an important advance in cancer therapy.

Lafarga et al. [403] evaluated in vivo on an animal model (rats) the toxicity of Dy-substituted (5 at.%) HA, synthesized by co-precipitation, and observed an increase of the oxidative stress indicators (i.e., lipoperoxides, nitric oxide) in the kidneys, lungs and liver, as well as a lower activity of the anti-oxidant enzyme (i.e., glutathione peroxidase). However, no significant change was observed in the membrane fluidity and adenosine triphosphate (ATP) activity. By functionalizing the HA nanoparticles with folic or glucuronic acid the toxicity could be significantly diminished. A MTT test with L929 mouse fibroblasts and aqueous HA injection into vitelline vein of egg indicated no toxicity of Dy-doped (10 at.%) HA used in doses of 25–500 µg mL^−1^ [392]. However, in this study performed by Tesch et al. [392] the real amount of Dy-doping evaluated by inductively coupled plasma mass spectrometry (ICP-MS) was approximatively half of the quantity theoretically inserted during the synthesis.

Erbium (Er) is a promising REM element due to its light emission and enhancement of biological properties of HA [372,404]. A strong and stable near-infrared emission at ~1540 nm, compatible with telecommunication applications, has been observed in Er-doped (~4.4 at.%) HA synthesized by co-precipitation [404]. Alshemary et al. [372] studied the in vitro bioactivity of Er-doped (2–10 at.%) HA fabricated by microwave-assisted precipitation from SBF solution, and showed the formation of a biomimetic apatite layer after 24 h of immersion.

Uranium (U)-doped HA with doping levels up to 10 at.%, remarkably did not alter MC3T3-E1 osteoblast viability and proliferation [375]. Further studies are necessary to understand the lack of toxicity when such an exogenous actinide metal is introduced in a controlled but large quantity into HA.

The nature of our exhaustive review of the literature allowed us to devise Table 3, which now encompasses, for the first time, the plethora of doping possibilities able to expand the biofunctional response of HA, with an emphasis on the role and the impact of each cation-doping species.

### 3.5. Cytotoxic Concentration of Cationic Species

Literature data on the actual cytotoxic cation release rates for this large variety of doped/substituted hydroxyapatite materials are rather scarce. Most researchers prefer to discuss the theoretical and/or experimental total cation dopant concentration [doping cation/(doping cation + Ca)] introduced into hydroxyapatite, and/or total dose concentration of doped-HA added to the cell media. When examining the dopants’ total content and their influence, contradictory cytotoxic levels are often signalled. This is to be expected since the cation release rate is governed by a series of factors such as crystallinity or particle morphology and size of the tested material, which are strongly dependent on dissimilarities in the chosen synthesis method, technological preparation recipes and post-synthesis processing stages. The authors advocate a more intimate understanding of the cation-substituted HA interaction with physiological media and cells, which can only be achieved by insightful studies (always including a control specimen of pure stoichiometric HA, comparing in the framework of a study the effect of more than one cation, focusing on the actual ionic release rates and not on the doped HA powder doses only). It is recommended for researchers to couple their bio-functional assays with determination of the temporal ion-release profiles, determined for instance by ICP techniques (with ppm/ppb sensitivity). Such systematic studies could help collate the prerequisite multiple and congruent demonstrations on the promise of a given cation and its optimal action dose, enable trustworthy conclusions, and allow for a reliable and safe transition of doped-HA from research bench-work to commercial and/or clinical applications, with great health and societal impacts.

**Figure 4:**
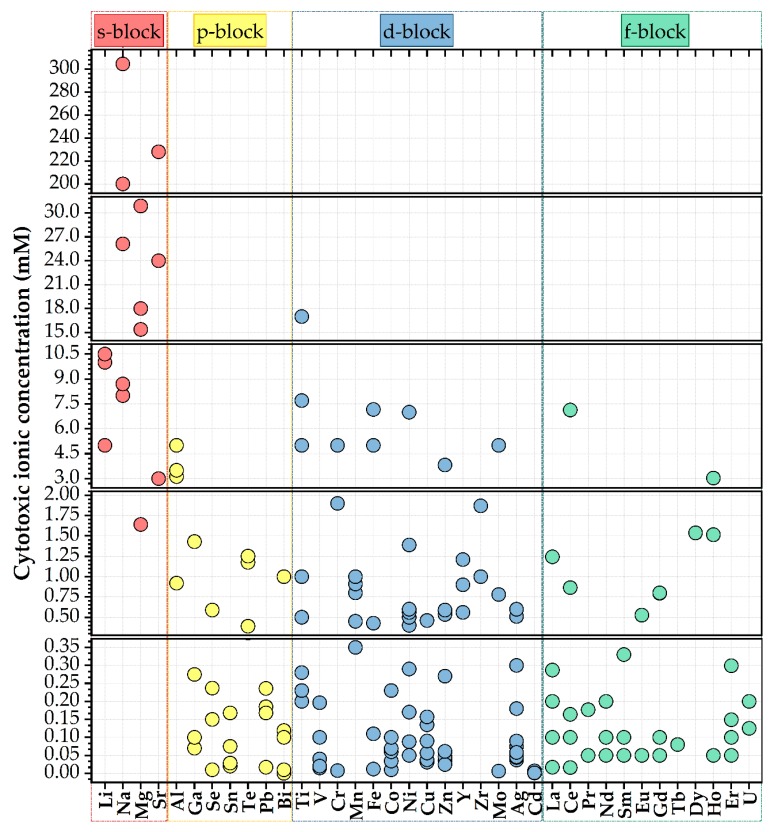
Half-maximal cytotoxic concentration (in mM) of various cationic species: Li [428,429,430], Na [431,432,433,434], Mg [435,436,437,438], Sr [439,440,441], Al [442,443,444], Ga [408,445], Sn [406,446,447], Te [448,449,450], Pb [427,451], Bi [452,453,454,455], Ti [406,408,443,444,456,457], V [408,442,443,444], Cr [408,442,443,444], Mn [443,458,459,460], Fe [426,442,443,461], Co [406,408,442,443,444,462,463], Ni [406,408,430,443,444,445,462,463,464,465], Cu [406,408,445,462,466], Zn [406,408,457,462,467], Y [468,469,470], Zr [406,442], Mo [442,443,471,472], Ag [406,408,445,471,473,474,475,476], Cd [408,462,471], La [477,478,479], Ce [406,480,481,482], Pr [480,483], Nd [480,484], Sm [480,485], Eu [477,480], Gd [480,486,487], Tb [488], Dy [485], Ho [480,485,489], Er [480,483,485], U [490,491].

Further scientific literature surveys were dedicated to the identification of cytotoxic concentration of the cations under scrutiny here, irrespective of their host material. The gathered information, presented in Figure 4, indicates a seemingly non-inhomogeneity of existing data. But this is only apparent because the cytotoxic concentration threshold which induced a cell’s growth decrease by 50% is dependent on a large palette of influential factors: source material of the ion [406,407,408], crystalline quality (a lower crystallized material will possess a higher free energy and ion molecular mobility, the consequence of which is accelerated degradation rates, and thereby faster release of active agents [409,410]), particle size (which influence the total active surface area) [411,412,413,414,415], shape/morphology of the particles (e.g., spheres, polyhedra, rods, platelets, random) [414,416,417,418,419], valance and oxidation state of the cation [420,421,422,423,424,425], cells line type, and incubation time [407,408,413,419,426,427].

## 4. Rigorous In Vitro Testing of Bioactive Materials

With the current regulations regarding in vivo studies (highly restrictive in the European Union) it is mandatory to identify in vitro protocols that would return reliable results, and reduce the number of materials suitable to enter in the in vivo studies to one or maximum two.

Thereby, when designing a medical device with enhanced properties (e.g., biomineralization capacity, osteoconductivity, angiogenic potential, or antimicrobial activity), the in vitro assay protocols need to allow a facile and trustworthy comparison between studies and results reported by individual research groups.

At the international level, there are a series of ISO standards that include recommendations concerning biological testing procedures and qualitative and quantitative evaluation markers. However, each of the ISO standards is dedicated to a definite material property, and employs a different type of testing media, with different degree of compositional complexity, used under various ambient conditions:

- **ISO 10993-14:2001**—*Biological Evaluation of Medical Devices—Part 14: Identification and Quantification of **Degradation** Products from Ceramics.*
  Medium for extreme tests: buffered ***citric acid solution***, pH = 3.0 ± 0.2 at a temperature of 37 ± 1 °C, in normal atmosphere;
  Solution for simulated tests: buffered ***tris(hydroxymethyl)aminomethane (Tris)-HCl solution***, pH = 7.4 ± 0.1 at a temperature of 37 ± 1 °C, in normal atmosphere.
- **ISO 16428:2005**—*Implants for Surgery—Test Solutions and Environmental Conditions for Static and Dynamic **Corrosion** Tests on Implantable Materials and Medical Devices.*
  Medium: *aqueous **solution of sodium chloride** (0.9% NaCl mass fraction) or **Ringer’s solution** isotonic aqueous solution of NaCl*, pH = 7.0 at a temperature of 37 ± 1 °C, in normal atmosphere.
- **ISO 16429:2004**—*Implants for Surgery—Measurements of Open-Circuit Potential to Assess **Corrosion** Behaviour of Metallic Implantable Materials and Medical Devices over Extended Time Periods.*
  Medium: *aqueous **solution of sodium chloride** (0.9% NaCl mass fraction)*, pH = 7.0 at a temperature of 37 ± 1 °C, in normal atmosphere. For more stringent test conditions, more acidic test solutions are recommended.
- **ISO 23317:2014**—*Implants for surgery*—In vitro *Evaluation for **Apatite-Forming Ability** of Implant Materials. (i.e., **Bioactivity/Biomineralization Capacity** Testing).*
  Medium: *Tris-buffered **simulated body fluid*** (*ionic concentration in mM:* 142.0 Na^+^, 5.0 K^+^, 1.5 Mg^2+^, 2.5 Ca^2+^, 147.8 Cl^−^, 4.2 HCO_3_^−^, 1.0 HPO_4_^2−^, and 0.5 SO_4_^2−^), pH = 7.4 at a temperature of 36.5 ± 0.2 °C, in normal atmosphere.
- **ISO 10993-5:2009**—*Biological Evaluation of Medical Devices—Part 5: Tests for* **in vitro *Cytotoxicity***.
  Medium: *culture medium (e.g., Dulbecco’s Modified Eagle Medium) with or without serum* such as to meet the growth requirements of the selected cell line, pH = 7.4 at a temperature of (37 ± 1) °C, in a humidified atmosphere of 5% CO_2_.
- **ISO 22196:2011**—*Measurement of **Antibacterial Activity** on Plastics and Other Non-Porous Surfaces.*
  Medium for suspension assays: ***nutrient broth*** *(containing meat extract, peptone, NaCl)*, at a temperature of (35 ± 1) °C and a relative humidity of not less than 90% for 24 ± 1 h, in normal atmosphere.

In vivo testing should never be considered before a thorough in vitro investigation.

We shall briefly discuss the positive aspects and the shortcomings of the most frequently used in vitro tests.

### 4.1. Biomineralization Capability (Bioactivity Testing)

The existence of a soundly crafted standard is welcomed in our opinion, since it can lead to meta-analyses and to facile comparisons between materials explored in different studies. However, a poorly and outdated standard can harm scientific endeavours and limit important and significant discoveries that can be safely transferred into medical practice.

For instance, the current in vitro standard for biomineralization testing (i.e., ISO 23317:2014) is, in our opinion, scientifically outdated, as it uses a purely inorganic solution (simulated body fluid, SBF), supersaturated towards the HA components, under normal atmospheric conditions [492]. It has the “advantage” of delivering fast and almost always positive results, even for materials otherwise widely-considered inert [147,493,494,495]. Many groups have started to acknowledge these limitations and are actively seeking a more reliable bioactivity assay [492,496,497,498,499,500], proving that even in complex organic–inorganic media, under the correct biomimetic conditions, biomineralization can be successfully, but rigorously, tested. 

For some of the ISO tests performed in purely inorganic media (e.g., ISO 10993-14:2001) it is recognized that supplementary biological factors, such as amino-acids, enzymes and proteins, can change the solubility/degradation rate of the material, and this it is not accounted for. Since the doping ion release would be different in each of these dissimilar media, it seems rather difficult to cross-examine results obtained by applying such standardized in vitro testing protocols. When studying the capacity of a biomaterial to induce a process, the in vitro protocol should mimic as much as possible the conditions that the implant would encounter in vivo. Although, the in vivo conditions are very difficult to be replicated with high fidelity in vitro, we should all acknowledge the acute need for improved and congruent protocols. As opposed to the SBF assay, which suggests a nearly universal biomineralization capability of materials, a test performed in complex media such as Dulbecco’s modified Eagle’s medium (DMEM)-like cell culture medium supplemented with serum (10%), produces biomineralization only for truly bioactive materials [496,497,498,499,500], being thus a reliable refinement tool for innovative material designs.

Only a rigorous and homogenous testing of bioceramics can filter the best material designs from abundant possibilities, and allow for a rapid translation to clinical applications. Thereby, we, along with an increasing number of researchers [497,498,499,500], would recommend advanced biomimicry testing of the biomineralization potential, using cell cultures media supplemented with 10% serum at 37 °C, in a humid atmosphere with 5% partial pressure of CO_2_ (as found in living tissues).

### 4.2. Degradation and Corrosion Tests

Three ISO standards (ISO 10993-14:2001, ISO 16428:2005, ISO 16429:2004) offer recommendations for such tests, but a series of issues persist:

- Using pure inorganic fluids for testing (i.e., citric acid, (Tris)-HCl, 0.9% NaCl, Ringer’s solutions) is not a viable choice because, as presented before, the organic component of the intercellular fluid interacts with the implant surface and greatly modifies the interactions with the biomaterial. The use of a suitable testing environment is of foremost importance since these specific material features (degradation rate and corrosion resistance) are dependent on the material surface properties and its ability to adsorb organic moieties, partial dissolution and the consequent ionic exchanges.
- In the attempt to compress the time needed for a degradation test and peek into the future, the ISO 10993-14:2001 standard uses buffered citric acid solution (at a pH = 3.0 ± 0.2) to force degradation. However, since this solution is only inorganic and with a pH value never to be encountered at the implantation site, results can significantly vary from the actual events that will occur in vivo for the tested material over the long-term.
- Such standards are designed mainly for testing bulk materials, and are focused on the weight of the specimen, not taking into account one of the most important parameters: the contact area with the fluid. The focus is on the ratio between the mass of specimen and volume of fluid, but systems to be studied differ a lot with respect to the interaction area per gram of substance. Pellets, scaffolds (with macro- and micro-porosity), powders with different particle size, and thin (or thick) smooth (or rough) films induce huge differences in the ratios between the mass of substance and the area of interaction with the testing medium. An overview of this particular matter along with a several proposals can be found in [492].

### 4.3. Biocompatibility Assays

Nowadays, the biocompatibility testing of a material with prospects for biomedical application, is mandatory. Different cell lines are used to assess cell proliferation, cell toxicity, capacity to induce bone matrix formation, and cell differentiation. The ISO 10993-5:2009 standard recommends for extract testing, direct contact, and indirect contact procedures testing periods of 24 h, at least 24 h, and between 24 and 72 h, respectively. The standard lays the ground for some basic biocompatibility tests, but its concepts might be considered somewhat outdated.

Cell proliferation can be evaluated by:

- Classic, **MTT** (3-(4,5-Dimethylthiazol-2-yl)-2,5-Diphenyltetrazolium Bromide), **MTS** (3-(4,5-dimethylthiazol-2-yl)-5-(3-carboxymethoxyphenyl)-2-(4-sulfophenyl)-2H-tetrazolium)), **XTT** (2,3-bis-(2-methoxy-4-nitro-5-sulphenyl)-(2H)-tetrazolium-5-carboxanilide) assays, that returns a value linked to general mitochondrial activity of the cells. Errors are given by different factors (e.g., differentiation of stem cells induces growth of mitochondria number per cell and increased activity). *Advantages:* simple and fast procedure, reliable results when working with homogenous terminally differentiated cells, cheap equipment and kits; *Disadvantages*: low reliability when working with heterogeneous cell cultures for differentiating experiments, indirect measure of proliferation;
- Quantifying double-stranded (ds) DNA by fluorescence (more ds-DNA, means more cells, ergo higher proliferation). Commercial kits are available. *Advantages*: direct measure of proliferation, very good and reliable results when working with heterogeneous cell cultures with many cell types (differentiation experiments), affordable equipment (98 well fluorescence reader), commercial kits are available; *Disadvantage*: complicated procedure;
- Cell counting when possible. *Advantage*: can be somewhat automated with a flow cytometer; *Disadvantages*: the classic counting technique uses microscopy, which is very laborious, time consuming, and impossible when dealing with a large number of situations (i.e., at least 10 microscopy fields per situation are required, with minimum 500 cells, numbered by three different examiners).

Cell toxicity can be evaluated by:

- Studying their morphology, when possible (as presented in ISO 10993-5:2009). This is a laborious method as it requires examination of a minimum 500 cells per situation acquired from a minimum 10 different randomly-chosen microscopy fields by three separate individuals. This renders the method almost impossible, when the experiment would involve a large number of materials;
- Measuring the LDH (lactate dehydrogenase) activity in the medium in which the cells were cultivated. LDH is an active intracellular enzyme found in all cells. Upon death, the cell releases this LDH into the medium and, therefore, this enzyme activity is proportional to the number of dead cells [501]. The method is easy to perform, fast, and returns reliable results on the same samples investigated for cell proliferation by mitochondrial activity tests;
- Measuring mitochondrial activity (MTT, MTS, XTT), as presented in the ISO 10993-5:2009 standard. It is a surrogate test for cytotoxicity: lower values with respect to control, due to lower general mitochondrial activity, are interpreted as results of cellular death, but this can also be an effect of slower proliferation values induced by the material. Thereby, it should not be used as stand-alone assay for cytotoxicity;
- Fluorescence apoptosis and cell viability kits (e.g., DAPI, annexinV, propidium iodide kit and Calcein AM/EthD-1 kit) are simple and widely used assays that provide good results, especially for flat substrates and examination with a confocal microscope. Calcein AM enters live cells and is converted in the cytoplasm in a green fluorescent compound, which does not exit from the cytoplasm. The dead cell nuclei have a red fluorescence due to EthD-1 that can penetrate only through the membrane of dead cells. As such, by fluorescence confocal microscopy the ratio of dead cells can be assessed. For 3D scaffolds it provides good results when the reading is done by a flow cytometer only, if the protocol recuperates and counts also the prior detached cells (which makes it a more difficult variant);
- Measuring the intracellular colorant uptake, as presented in the ISO 10993-5:2009 standard. The procedure is time consuming, but offers reliable results.

For biocompatibility we would suggest using a LDH activity kit in conjunction with a MTS/XTT/MTT assay in order to counteract the errors that may occur from high proliferation coexistence with high apoptosis.

The shape of the material to be tested can vary and can pose great challenges. The easiest situation for cell culturing is constituted by dense and smooth coated specimens. In the case of bulk material specimens, flat surfaces (to be obtained by polishing) with same area are needed as well. 

The most problematic situation is represented by the porous scaffolds, produced by various additive manufacturing techniques. Frequently these types of testing samples have two degrees of porosity: (*i*) micro-porosity generated by elimination of substances needed to produce the ink or the carrying thermoplastic filament compounds; and (*ii*) a macro-porosity resulting from the designed spacing between the individual material rods, which is usually situated in the range 100–500 µm. 

The challenge arises from the difficulty of uniformly seeding a certain number of cells into the scaffold. The detached cells have a diameter of ~15 µm and fall through the rods of the scaffold to the bottom of the well. Also, when comparing scaffolds with different macro-porosity (different spacing between the rods) the problem becomes even more complicated, as the available space in the scaffold is different.

We did not find in the literature a protocol that would allow the seeding of identical numbers of cells in scaffolds with different macro-porosity.

### 4.4. Osteoinduction Ability

In order to enhance bone healing, few methods to boost osseointegration, cytocompatibility and bone matrix production were envisioned. As such along with incorporation/adsorption of growth factors that stimulates osteoblastic lineage [502,503,504], some cation doping into HA was also extensively investigated, as was shown in the previous sections of this review.

The most common protocols to assess the formation of new bone matrix involve the alizarin red technique. However, when cells are grown on opaque substrates (such as titanium) that do not allow bright-field microscopy assessment, other means of investigation must be searched for.

One other solution is to quantify the proteins (i.e., collagen, ostein, osterix, osteopontin) present in the matrix formed by the cells, by immunofluorescence techniques. Quantification of such protein and cell solubilisation markers by enzyme-linked immunosorbent assay (ELISA) or the Western blot method is more difficult, but delivers better results.

### 4.5. Cell Differentiation Capacity

In order to boost bone healing, a great number of osteoblasts is needed, and the simplest solution would consist in the ability of the implant material to boost stem cell differentiation toward osteoprogenitor cells and osteoblasts.

Nowadays, seemingly the most desired property of HA materials is the capacity to induce stem cell differentiation. In the quest to obtain fast healing, the scientists could fall into the grave error of inducing the disappearance of the stem cell pool, and thereby generating a high risk of implant failure in the long-term. Indeed, in a study performed by Popa et al. [15], it was shown that bone is a complex organ subjected to continuous remodelling processes, and if inducing a rapid large-scale differentiation of stem cells, a great number of osteoprogenitor cells would be produced, but, after a period of time, the osteoprogenitor cells will cease to exist as a consequence of the stem cell pool absence.

The generation of osteopotent daughter cells in sufficient number to induce healing, is a complex phenomenon which should be reserved to the cell signalling between stem cells and their niche and inflammatory cells present at implantation site/wound. The stem cells pool, along with its niche and the complex signalling processes are a micro-universe yet to be fully understood. The authors advise fellow researchers to act with caution when integrating biomaterials that possess the ability to force abrupt stem cell differentiation. In the absence of long-term in vivo evidences on the biological outcome of such materials, awareness/caution on the risks of meddling with stem cell signalling is in our opinion still necessary.

Osteogenic differentiation can be studied using immunofluorescence for markers specific to osteoblastic lineage such as: extracellular matrix proteins (e.g., collagen, osteopontin, osteocalcin, bone sialoprotein) or intracellular proteins which have enzymatic function (e.g., alkaline phosphatase) [505]. The cells marked with fluorochromes can be viewed with an epi-fluorescence or confocal microscope. Production of bone by osteoblasts could be investigated also by cyto-chemistry methods (e.g., alkaline phosphatase activity assay). More complex techniques, that are not at hand for most scientists due to the high cost of equipment and kits, are transcriptomics, proteomics and metabolomics. An extensive survey of the RNA profile of the cells by DNA microarray or real-time polymerase chain reaction (real-time PCR) can confer clarity regarding the stage of cell differentiation and the mechanism of this process [506].

The von Kossa assay produces a black-grey stain where Ca deposits are located in the tissue by replacing Ca ions with silver ones. Therefore, the von Kossa assay could be only applied for in vitro tests that incubate osteoprogenitor cells with cell-growing medium that was in contact with the powders/scaffolds of calcium phosphates (HAs included).

### 4.6. Pro-Angiogenic Properties

The capacity to induce angiogenesis is envisioned as an important trait of a scaffold, since it will help healing by generating a sufficient blood supply for the cells that will colonize the implant. For metallic implants coated with HA there is no need for a pro-angiogenesis property, since the healing will take place only on a surface and the normal bone around the implant will produce all the blood vessels it needs.

The cells that will produce new blood vessels derive from stem cells through proliferation towards an angiogenic fate. The angiogenic lineage results from the modifications in a constellation of signalling molecules: pro-inflammatory cytokines, interleukins, stem cell factor, Notch, Vascular endothelial growth factor (VEGF), epidermal growth factor (EGF), fibroblast growth factor (FGF), platelet derived growth factor (PDGF), insulin-like growth factor (IGF), tumour necrosis factor alpha/beta (TNF), transforming growth factor beta 1 and 2 (TGF-β1 & TGF-β2), or Wnt [507,508].

One possibility for generating blood vessels in the scaffold is to use recombinant pro-angiogenic growth factors such as, VEGF, PDGF, IGF, or TGF-β1 & TGF-β2 adsorbed into the scaffold [502,509]. These proteic substances are difficult to manage and manipulate, expensive and would require a complex authorization and quality control. Therefore, some ion additions (e.g., Li, Co, Ni, Mg, Sr, La) in the HA scaffold have been envisaged as a simple and cheap alternative [222,328,340,510,511]. To date, more than 120 articles have been published on this topic. Some of these studies reached an in vivo phase with encouraging results [512,513,514,515,516]. However, it is important to use a doping concentration that generates the desired angiogenic effect, but does not exhibit toxic side effects on a long-term ion release. In vivo angiogenesis studies typically witness micro-vascularisation formation, along with growth of blood vessels with a greater diameter. However, questions about the long-term adverse side-effects on the healing process, such as the generation of too many blood vessels and the out-growth of the implant, need to be addressed as well in the future.

Furthermore, numerous groups found that macro-porosity greatly influences the vascularisation of ceramic scaffolds. While a pore dimension over 30–40 μm can enable endothelial cells to enter the scaffold [517,518], larger pore sizes (>150 μm) facilitate the development of blood vessels with greater diameter and total volume, upon in vivo implantation [519]. This effect is observed until a pore size of 500 μm, where the plateau of blood vessels diameter and volume is reached [520,521,522].

In vitro pro-angiogenic properties investigations can be carried out on endothelial cell cultures, as the viability, proliferation, and cytotoxicity of such cell lineages is basic.

More complex experiments would imply (*i*) measuring of the amount of pro-angiogenic factors secreted by these cells when cultivated in the presence of HA materials, or (*ii*) RNA quantification of the activation of the proangiogenic genes.

### 4.7. Antimicrobial Activity

Most studies determine the antimicrobial activity on both Gram-positive and Gram-negative bacterial strains, having as major exponents *S. aureus* and *E. coli*. These bacteria are the most frequently met in implant infections. 

The ISO 22196:2011 standard provides a protocol for antimicrobial testing of bulk and thin films of biomaterials with nutrient broth and *S. aureus* or *E. coli*. There are some limitations:

- The tested material should be flat and compact with a surface of minimum 6.25 cm^2^, of which 4 cm^2^ should be reserved for bacterial interaction;
- Various types of nutrient broth have been observed to interact differently with the biomaterials, causing a variety of degradation rates, and therefore dissimilar antibacterial activities;
- Because of their nature and geometry, powders and 3D scaffolds with macro- and micro-porosity, cannot be tested according to this ISO standard protocol. Therefore, adaptive measures should be devised.

For powders:

○ A nutrient media powder suspension is inoculated with a known number of colony-forming units (CFU) to a final concentration of around 10^5^−10^6^ CFU mL^−1^, under continuous agitation in an incubator at 37 °C for a desired period of time. The number of bacterial cells that remained viable (viable cell count, VCC) is to be investigated by serial dilutions from each situation and seeding on simple agar plates (in an analogue manner to the ISO standard protocol);
○ Colorimetric or fluorescence tests can be performed on samples, and rapid results are obtained based on previous control measuring curves established for each type of bacteria (e.g., MTS/XTT, cresyl violet, fluorescein diacetate). The fluorescence techniques use more expensive reagents and readers, but their measurement is more reliable since turbidity of the sample generated by powder material dissolution does not affect the reading. Fluorescein diacetate is used in a standard method for the assessment of water contaminated with microorganisms and could be considered very reliable.

For 3D scaffolds:

○ The scaffold would require an incubation in a given volume of nutrient media inoculated with a known number of CFU;
○ Antimicrobial activity of a 3D structure is very hard to investigate because not all the bacterial cells can be harvested, since some of them could be very strongly adhered inside the scaffold, and therefore hard to detach;
○ After the desired testing period, since the bacterial cells could be adhered inside the scaffold and cannot be reached, only a reading of a soluble coloured/fluorescent product of bacterial metabolism can provide insights. Some materials absorb coloured substances and make such tests impossible to carry out.

## 5. Future Perspectives: Co-Substituted Hydroxyapatite Bioceramics

Nowadays, as we enter the era of personalized medicine, the design of a successful implant implies making the right compromises with respect to the material formulation, shape, structure, mechanical performance, biocompatibility, pro-angiogenic or pro-osteogenic properties, and wide-range antimicrobial activity, so as to aptly and comprehensively respond to the patient’s problem. Starting with natural cation and anion doping (Na, Mg, Sr, carbonates, Cl, F) and the trace elements (Zn, Cr, Co, Mn, Si) that are naturally found in human bones [5], the synthesis of co-substituted HA could pave a way toward the design of implants with combined multi-biofunctionality.

In the realm of antimicrobial efficiency, multiple doping with low ionic contents in HA seems to be the key to achieving potent activity, capable of combating the resilience of the microorganisms even adapted to conventional antibiotics, while limiting the toxic side effects. The simultaneous release of ions with different action mechanisms could enable not only preventing the adaptation of bacteria and fungi, but also widening the antimicrobial range against more pathogen strains. 

In this respect, we acknowledge the recent contributions focused to ascertain both synergic ion doping combinations and their optimal concentration. So far, HA has been co-substituted with: Ag/Bi [523], Ag/F [524], Ag/Mg [525,526], Ag/Si [527], Ag/Sr [528,529], Ag/Zn [281,530], Ce/Fe [381], Ce/Eu [531], Sr/Ce [344,532], Sr/Cu [533], Sr/Zn [534,535], Zn/Cu [533,536], Zn/F [78], Zn/Fe [537], La/Ag [405], La/Cu [538], Sm/Gd [354], Tb/Gd [539], Ce/Zr/F [540], Ag/Ti/F [541], Mg/Zn/Co [542], Sr/Co/F [543], Ag/Cu/Zn/F [544], or Ag/Cu/Zn/Ti [545].

HA-based medical devices could also be coupled with cell therapy, enhancing their short- and long-term performances. In this respect, promising results were obtained for autologous stem cells, osteoprogenitor cells, or bone marrow aspirates. Quarto et al. [546] have used with clinical success autologous stem cells to induce healing of large defects of long bones. Scaffolds of biomaterials cultivated with autologous osteoprogenitor cells, implanted to boost bone healing, have shown good results up to 7 years [547]. Bone marrow aspirates, “a cocktail” of stem cells, niche cells and differentiated cells, were also used in clinical studies with success rates of 80–90% to heal delayed or non-unions of long-bone fractures [548,549,550,551].

In the light of the numerous contradictions, signalled in this review, a more rigorous and systematic scientific approach is recommended, studying compositional series of substituted HA samples, avoiding splitting the research into a number of manuscripts, and always coupling the antimicrobial effect demonstration with cytotoxicity assays and determinations of the actual temporal release profiles of the therapeutic ions.

## Figures and Tables

**Table 1:** Preparation of hydroxyapatite (HA) from natural resources.

Elements	Source	Synthesis Method	Refs.
Bovine	Cortical bone	**Pre-cleaning**: (*i*) removal of soft tissue; (*ii*) cut into small pieces and boiled in water for 2 to 3 h; (*iii*) dry in an oven at 80 °C for 72 h; (*iv*) crush and subsequently grind by ball milling for 24 h. **Heating**: calcination in a furnace at temperatures in the range of 600–1100 °C, for 3 h, with heating and cooling rates of 5 °C min^−1^.	[93]
	Cortical bone	**Pre-cleaning**: (*i*) removal of soft tissue; (*ii*) crushing and milling process. **Heating**: sintering in a furnace at 1200 °C for 2 h or 4 h, with a heating and cooling rate of 5 °C min^−1^ and 10 °C min^−1^, respectively.	[92]
	Teeth	**Pre-cleaning**: (*i*) removal of soft tissue; (*ii*) removal of remnant impurities by mechanical scraping; (*iii*) boiling in distilled water for 30 min; (*iv*) repeated the aforementioned steps three times; (*v*) drying in the sun for 3 days. **Heating**: (*i*) calcination in humid atmosphere at 735 °C for 1 h with a heating rate of 7 °C min^−1^; (*ii*) sintering at 1150 °C for 1 h with a heating rate of 7 °C min^−1^.	[108]
Pig	Cortical bone	**Pre-cleaning**: (*i*) hot water treatment; (*ii*) removal of organic compounds by scraping; (*iii*) de-proteinization in a boiled mixture of 1 M NaOH and 1 M HCl at 100 °C for 5–10 min; (*iv*) dried in an oven at 100 °C overnight; (*v*) crushing and grinding. **Heating**: calcination in air at 600 °C, 800 °C or 1000 °C at a heating rate of 5 °C min^−1^ followed by cooling to room temperature.	[99]
	Cortical bone	**Pre-cleaning**: (*i*) removal of soft tissues and fluids; (*ii*) boiling bone slices at 154 °C, 4 atm; (*iii*) drying in vacuum; (*iv*) milling; (*v*) removal of remnant fat and protein moieties by hydrothermal process. **Heating**: (*i*) calcination at 5 °C min^−1^ at 600 °C (allows the decomposition of organic tissue); (*ii*) sintering at 1000 °C (induces physico-chemical changes) from 1 to 50 h; (*iii*) cooling in the furnace in air.	[100]
Camel	Cortical bone	**Pre-cleaning**: (*i*) removal of organic compound; (*ii*) dry-heating at 100 °C for 1 h; (*iii*) cut in small pieces and immersion in acetone for 1 h. **Heating**: calcination at 1000 °C for 3 h at a heating rate of 10 °C min^−1^, and then slowly cooled down to room temperature.	[102]
Sheep	Cortical bone	**Pre-cleaning**: (*i*) removal of femoral heads; (*ii*) de-proteinization with NaOH; (*iii*) washing and drying. **Heating**: (*i*) calcination at 850 °C for 4 h in air; (*ii*) crushing and milling.	[103]
	Dentine	**Pre-cleaning**: cleaning and washing the teeth. **Heating**: (*i*) calcination at 750 °C for 5–6 h; (ii) separation of dentine from enamel; (*iii*) ball grinding; (*iv*) sintering at 1000–1300 °C for 4 h.	[109]
Chicken	Egg-shells	**Pre-cleaning and synthesis**: (*i*) crushing egg-shells; (*ii*) simultaneous removal of organics and transformation of CaCO_3_ into CaO by calcination at 900 °C for 1 h; (*iii*) addition of water and phosphoric acid; (*iv*) precipitation overnight, followed by filtration and washing; (*v*) drying the HA product at 60 °C for 24 h. **Heating**: sintering in air (after sieving and pressing) at 900–1300 °C for 1 h, with a heating rate of 10 °C.	[110]
Fish	Bones	**Pre-cleaning**: (*i*) removal of organic compounds by brushing and then boiling at 100 °C for 10 min; (*ii*) drying at 90 °C for 100 min and then crushing to powder; (*iii*) de-proteinization by reflux method using a 5% KOH solution. **Heating**: sintering at 600–1000 °C.	[111,112]
Mussel	Shells	**Pre-cleaning and synthesis**: (*i*) mechanical cleaning and calcination in air at 1300 °C for 6 h (*ii*) Rathje fabrication method: mixing seashells powder with water and H_3_PO_4_ with magnetic stirring during the synthesis for 2 h at 700 rpm; (*iii*) filtering, followed by drying at room temperature for 168 h, and then at 100 °C for 24 h. **Heating**: sintering at 1200 °C for 10 h.	[57]
Snail	Shells	**Pre-cleaning and synthesis**: (*i*) thoroughly cleaning of sand particles and other foreign materials; (*ii*) drying, crushing into small particles, ball-milling; (*iii*) sieving; (*iv*) mixing the as-obtained CaCO_3_ powder with water and H_3_PO_4_ solution, followed by continuous stirring at 80 °C for 8 h; (*v*) drying at 100 °C overnight in an incubator. **Heating**: calcination at 800 °C for 4 h in air.	[63]
Cuttlefish	Whole	**Pre-cleaning and synthesis**: (*i*) cutting into small pieces; (*ii*) heat-treatment at 110, 500, 1000 °C with a heating rate of 5 °C min^−1^; (*iii*) mixing the as-obtained CaCO_3_ powder with an aqueous NH_4_H_2_PO_4_ solution to a Ca/P molar ratio of 1.67; (*v*) drying at 200 °C for 1–72 h, using heating and cooling rates of 5 °C min^−1^.	[60]
Dolomic marble	Origin: Ruschiţa, Romania	**Pre-cleaning and synthesis**: (*i*) mechanical cleaning and calcination in air at 1300 °C for 6 h (*ii*) Rathje fabrication method: mixing seashells powder with water and H_3_PO_4_ with magnetic stirring during the synthesis for 2 h at 700 rpm; (*iii*) filtering, followed by drying at room temperature for 168 h, and then at 100 °C for 24 h. **Heating**: sintering at 1200 °C for 10 h.	[57]
Red algae	Whole	**Pre-cleaning**: (*i*) rinsing at high-pressure; (*ii*) drying at room temperature for 24 h; (*iii*) sieving; (*iv*) thermal treatment to burn-off the organic material, at 650–700 °C for 12 h, with a low heating rate of 0.5 °C min^−1^ to prevent decomposition of algae; (*v*) alkalinisation with ammonium hydroxide at ambient pressure and 100 °C for 12 h under continuous stirring at speed of 100 rpm; (*vi*) filtration and neutralisation by repeating washing and drying overnight at 90 °C. **Heating**: thermal treatment at 60 °C, 105 °C, 450 °C, 550 °C and 1000 °C for 1 h each.	[113]

**Table 2:** Other fields of applications for cation-substituted hydroxyapatite.

Cation Dopant	Field of Application [Refs.]
Na	Sensors [162]; Catalysis [163]
Sr	Catalysis [164]
Ba	Water decontamination [165]; Catalysis [166]
Al	Environment decontamination [167,168,169]; Catalysis [170]
Sn	Radionuclides and heavy metals scavengers (decontamination) [171]
Pb	Catalysis [172,173]
Y	Electrochemical devices [174]
Ti	Catalysis [175,176,177]
V	Catalysis [178]
Mn	Catalysis [179]; Optoelectronics [180]
Fe	Sensors [181]; Catalysis [182,183]
Co	Sensors [184]
Ni	Catalysis [185,186,187,188]
Pd	Catalysis [189,190]
Pt	Catalysis [191,192]
Cu	Catalysis [193,194,195,196]; Water decontamination [197]
Ag	Catalysis [198,199,200]
Au	Catalysis [194,201,202]
Zn	Catalysis [203,204,205]
Sm	Optoelectronics [206]
Eu	Optoelectronics [206,207]; Environmental [208]
Gd	Optoelectronics [206]
Tb	Catalysis [209]; Optoelectronics [210]
Dy	Optoelectronics [211]

**Table 3:** Synopsis of the bio-functionality realm of cation-substituted hydroxyapatites.

Cation (M)	Sample Form	Doping Range [M/(M + Ca)]∙100 (at.%)	Bio-Functionality/Effect of the Dopant	Refs.
**Li**	Powder Scaffold Coating	0.5–2	○ Stimulates in vitro bone-like apatite growth in simulated body fluid (SBF); ○ **In vitro cytocompatibility** with bone marrow *mesenchymal stem cells (BMMSCs)*, *calvaria isolated osteoblasts*, *human osteosarcoma (MG63)* cell lines; ○ Increases cell viability and proliferation; ○ Li-HA scaffolds revealed in vivo (Japanese white rabbits) good **osteogenesis** and **angiogenesis** potential; ○ Improves the **compressive mechanical strength**; ○ Induces the **new bone formation in animal model**.	[98,221,222,223,224]
**Na**	Powder Coating	5	○ Enhances the in vitro biomineralization of apatite in SBF; ○ **In vitro cytocompatibility** with *mouse skull osteoblastic cell (MC3T3-E1)* lines; ○ Promotes **osteoblast proliferation**; ○ Increases **coating adhesion** on reinforced carbon fibres; ○ **Stimulates dense new bone formation** in animal model.	[227,229]
**K**	Powder	2.5–47	○ The **adsorption of bovine serum albumin** increases with dopant concentration; ○ Constitutes a **potential** needle-free **protein/antigen trans-dermal delivery** system.	[232,233]
**Mg**	Powder Coating	1–53	○ Mg doping stimulates bone-like apatite growth in SBF; ○ **In vitro cytocompatibility** with *MC3T3-E1*, *MG63*, *primary rat osteoblasts (rOBs)* and *endothelial cells (rECs)*; ○ Improves the **adhesion** and stimulates the **proliferation and differentiation** of osteoblasts; ○ Mg **(~1 at.%**) induces **apoptosis of human monocytes**; ○ **Antibacterial effect** against *S. aureus* (ATCC 29213), *E. coli* (ATCC 25922), and *P. aeruginosa* (ATCC 27853); ○ **Enhances osteoconductivity** as demonstrated **in vivo** on animal model.	[235,236,237,238,239,240,242,243]
**Sr**	Powder Coating	1–40	○ Improves the biomineralization capacity (both in SBF and modified Eagle’s medium (MEM) media); ○ **In vitro cytocompatibility** with *MG63*, *human foetal bone—cloned osteoblast (OPC1)*, *MC3T3-E1*, *human osteoblast (hFOB 1.19)* cell lines; ○ Enhances the osteoblast cells **proliferation** and **differentiation** (enabling new bone formation); ○ Inhibits osteoclast production and proliferation (reducing bone resorption); ○ **Enhances new bone formation**.	[240,243,244,245,246,249,250,251,252,255,257]
**Ba**	Powder	0.5–2	○ Increases the biomineralization capacity of CaPs in SBF. ○ No information on in vitro or in vivo biocompatibility; ○ **Does not inhibit the proliferation of pathogens** such as: *S. aureus* (COWAN 1), *Bacillus megaterium* (DMS 32), *E. coli* (ATCC 259225), *K. pneumonia* (FMC 5), and *C. albicans* (FMC 17).	[261,262]
**Al**	Powder	0.5–2.5	○ **In vitro cytocompatibility** with *mouse fibroblast cell (L929)* line.	[263]
**Ga**	Powder	n/a	○ **In vitro cytocompatibility** with *murine cells (RAW264.7)*; ○ **Antibacterial effect** against P*. aeruginosa (*MW1); ○ Non-effective against *E. coli* and *S. epidermidis*.	[264,266]
**In**	Powder	1; 3	○ **In vitro cytocompatibility** with *human limb tissue osteoblast* cells line (ATCC CRL-11372); ○ Improves the **osteoblasts’ adhesion and differentiation**; ○ Induces **certain levels of** *blood monocyte* **apoptosis**.	[238,267]
**Bi**	Powder	5–25	○ Increases HA dissolution rate; ○ Induces the in vitro formation of bone-like apatite in SBF; ○ **In vitro cytocompatibility** with *human limb tissue osteoblast* cells from (ATCC CRL-11372); ○ Improves **osteoblast adhesion** and **differentiation**; ○ Bi-HA (scaffolds)—polyurethane (matrix) elicit excellent **mechanical, biocompatible and osteoconductive** properties **in vivo** (rabbits); ○ **Antibacterial effect** against *S. aureus* and *E. coli*;	[267,268,269]
**Te**	Powder	0.04–0.22	○ **Antimicrobial effect** against *S. aureus*, *Bacillus subtilis*, *Micrococcus sp.*, *P. aeruginosa*, *Klebseilla sp.*, *Proteus mirabilis*, *Shigella dysenteriae* and *C. albicans*.	[272]
**Ag**	Powder Scaffold Coating	0.5–5	○ Induces the in vitro biomineralization of biomimetic apatite layers in both SBF and McCoy media; ○ **In vitro cytocompatibility** with *L929* (at Ag concentrations **<3 at.%**), *hFOB 1.19* (induces premature apoptosis, delayed differentiation or cell death at high Ag contents (>3 at.%) [250], but at low Ag concentrations (~0.5–2 at.%) sustains the cell functions without interference [280]), *human embryonic palatal mesenchymal (HEPM)* (Ag = 2 at.%) [273]) cell lines; ○ In vivo evaluations on Sprague-Dawley rats showed efficiency against methicillin-resistant *S. aureus* (MRSA) strains, while not producing argyria, or any other kind of skin disorder or affecting the brain, kidneys, liver or spleen of the animals [283]; ○ **Antibacterial effect** against: MRSA (UOEH6), *S. aureus* (ATCC6538, Cowan I, 0364, ATCC 25293), *S. epidermidis* (ATCC 35984), *Enterococcus faecalis* (ATCC 29212), *P. aeruginosa*, *Bacillus megaterium* (DMS 32), *E. coli* (ATCC25922, ATCC25923), *Klebsiella pneumoniae* (ATCC4352, 2968, ESBL, FMC 5), *Enterobacter cloacae* (61R), *Providencia stuartii* (1116), and *Citrobacter freundii* (1748); ○ **Antifungal effect** against yeast strains: *Candida krusei* (963) and *C. albicans* (FMC17); ○ Not effective as antibiotic against: *Bacillus subtilis* [275] and *Serratia marcescens* (0804) [276]; ○ Ag^+^ does not affect the densification of HA; ○ At low concentrations it decreases HA solubility; ○ Hardness is affected by Ag doping.	[250,261,273,275,276,277,278,279,280,281,282,283,284,285,286]
**Zn**	Powder Coating	0.1–50	○ Excellent in vitro bioactivity in SBF; ○ **In vitro cytocompatibility** with *MC3T3-E1*, *MG63*, *mouse Balb/c 3T3 clone A3 fibroblast* cell lines; ○ No inflammatory effect; ○ **Positive effect on osteoblast cells viability, adhesion, spreading, proliferation and differentiation**; stimulates **osteogenic activity and bone growth or healing**; ○ **Cytotoxic to** *human hepatocarcinoma (HepG2)* cells at concentrations **<1 at.%**, function of particle morphology; ○ **Antibacterial effect** activity against: *S. aureus* (CECT 976, ATCC 25923, ATCC 43300), *MRSA*, *S. epidermidis* (ATCC 14990), *Bacillus subtilis* (ATCC 6051), *S. mutans* (ATCC 25175), *Lactobacillaceae, Streptococcus sobrinus*, *E. coli* (CECT 434, MG1655, ATCC 12435, ATCC 25922), and *Enterobacter aerogenes* (ATCC 13048); ○ **Antifungal effect** against *C. albicans* (ATCC 10231); ○ **Enhances new bone formation** as demonstrated **in vivo** on animal model.	[66,238,243,274,277,281,282,287,288,289,291,292,293,294,295,296,297,298]
**Cu**	Powder Coating	0.04–5	○ Excellent in vitro bioactivity in SBF. ○ Beneficial for inducing **protein absorption, osteogenic differentiation, bone-like apatite nucleation** and **growth** at implant site; ○ **In vitro cytocompatibility** with *MC3T3-E1* and *rat calvarial osteoblast* cell lines; ○ A doping concentration of **1 at.% is cytotoxic** to *Balb/c 3T3 clone A3* mouse fibroblasts and to *HFOB 1.19* cellular lines, reducing the cells viability; ○ A significant level of **apoptosis** is recorded for a concentration of **1 at.%** for the *human monocytes* isolated from blood; ○ **Antimicrobial effect** against *S. aureus* (ATCC 25923) and *E. coli* (ATCC 25922); ○ **Antifungal effect** against *C. albicans* (ATCC 24433).	[238,299,300,301,302,303]
**Mn**	Powder Coating	0.4–20	○ Possesses the ability to induce the in vitro growth on biomimetic apatite in SBF; ○ **In vitro cytocompatibility** with *MC3T3-E1* and *hFOB 1.19* cell lines; ○ Stimulates cell **viability** and **proliferation**, and **improves metallic implant biocompatibility**; ○ Increases **bonding strength** between HA coating and metallic (Ti) implant; ○ Enhances the **corrosion resistance**.	[304,305,306,307,308,309]
**Fe**	Powder	1–50	○ **In vitro cytocompatibility** with *MC3T3-E1*, *hFOB 1.19*, *MG63* cell lines; ○ Increases **osteoblasts adhesion and proliferation**; ○ Fe^3+^ is involved in **osseointegration**; ○ **Not cytotoxic for doping levels <12 at.%**; ○ Induces **certain levels of** *human blood monocyte cells* **apoptosis**; ○ Fe-HA has **a great potential** as heating mediator in **hyperthermia therapy** of cancer, showing a fast and effective effect on hepatic and colon cancer; ○ **Antibacterial effect** against *S. aureus* and *E. coli*; ○ **Promotes bone-like apatite nucleation** both in vitro in SBF and in vivo in animal model.	[308,309,310,311,312,313,314]
**Ti**	Powder Coating	1–13	○ Induces in vitro formation of biomimetic apatite in SBF; ○ **In vitro cytocompatibility** with *rat bone marrow stromal*, *HFOB 1.19* (up to **~13 at.%**), and *MG63* cell lines; ○ Enhances cell proliferation, differentiation in osteoblasts and matrix mineralization; ○ **Antibacterial effect** against *E. coli* (IFO 3310); ○ Hardness and elastic modulus increases with Ti addition.	[68,177,301,316,317,318]
**Cr**	Powder	0.5–2.5	○ **In vitro cytocompatibility** with *L929* and *cervical cancer (HeLa)* cell lines up to a concentration of 800 μg mL^−1^; ○ **In vitro haemocompatibility** in the case of low doping concentrations (**~0.5 at.%**); ○ Cr-**HA does not exhibit mutagenicity** on *Drosophila melanogaster* Meigen larvae.	[319,320]
**Co**	Powder	0.2–27	○ **In vitro cytocompatibility** with *MG63* cell line; ○ Elicits **proangiogenic** and **osteogenic properties**; ○ **No haemolytic effect** for doping levels **up to 37 at.%**; ○ Might induce *human blood monocyte* cells apoptosis; ○ **Antibacterial effect** against *S. aureus*, *M. luteus*, and *S. flexneri*; ○ Ineffective against *P. aeruginosa* bacterial stain; ○ **Stimulates the osteogenesis** as demonstrated by **in vivo** tests on animal model.	[238,321,322,323]
**Ta**	Powder	0.13–0.27	○ **In vitro cytocompatibility** with *hFOB* cell line; ○ Promotes **osteoblast proliferation**.	[324]
**Ni**	Powder	0.8–8.3 (theoretical)0.2–2.4(determined by ICP-OES)	○ **In vitro cytocompatibility** with *MG63* cell line; ○ **Increases osteoblasts viability, proliferation and differentiation**; ○ **Antibacterial effect** against *E. coli* (ATCC 25922) and *P. aeruginosa* (DSM50071), when tested in combination with other dopants.	[328,329]
**Mo**	Powder	0.05–5.2	○ **Antibacterial effect** against *S. epidermidis* and *E. coli*; ○ **Antifungal effect** against *C. albicans*; ○ Enhances HA’s ability to **absorb the electromagnetic gamma radiation**.	[330]
**Y**	Powder Coating	1.3–7	○ **In vitro cytocompatibility** with *human osteoblast cells from limb tissue* (ATCC CRL-11372); ○ Stimulates **osteoblasts adherence and proliferation**; ○ Can be used for radioactive synovectomy to **treat haemophilic synovitis**.	[267,331,332,333]
**Cd**	Powder	n/a	○ **High toxicity** on zebra fish, which died after Cd-Ha exposure; ○ Toxic effect on the growth of plants.	[334,335]
**W**	Powder	0.7–32.3	○ **Catalytic activity** by enhancing the biosorption and adsorption of methyl orange by *E. faecalis* bacteria and further decolourization and removal from waste water; ○ **Increases gamma radiation absorption**, which makes it useful in radiation shielding.	[336]
**Hf**	Powder	0.5–15	○ **Cytotoxic to A549 human adenocarcinoma alveolar epithelial cells**, when Hf-HA is used **in combination with ionizing radiation** (photodynamic therapy); ○ In vivo (mice) tests show **tumour reduction** after using ionizing radiation and Hf-HA nanoparticles.	[339]
**La**	Powder Coating	2–30	○ **In vitro cytocompatibility** with *MC3T3-E1* and *L929* cell lines; ○ **No cytotoxicity** for adenocarcinoma (MCF-7) and human embryonic kidney HEK cells at a doping level of **2 at.%**; ○ **Antibacterial effect** against *S. aureus* (e.g., ATCC 25175), *E. coli*, *P. aeruginosa,* and *Bacillus*; ○ Improvement of mechanical properties: bonding strength and Vickers hardness.	[314,340,342,379,380,405]
**Ce (3+)**	Powder Coating	4–20	○ Induces the in vitro formation of bone-like apatite in SBF; ○ **In vitro cytocompatibility** with *L929* (for Ce-HA **dose <100 µg mL^−1^**) and *MC3T3-E1* cell lines; ○ **Cytotoxicity on pulmonary** *adenocarcinoma (A549)* cells in Ce0.1HA, but **improvement of cell viability** in conjunction with strontium [344]; ○ **Antibacterial effect** against *S. aureus* (ATCC 6538), *Lactobacillus* (ATCC 393), *E. coli* (8099), and *P. aeruginosa*; enhanced zone inhibition is achieved for Gram-negative *E. coli* with respect to Gram-positive *S. aureus*.	[344,346,377,382,383,384]
**Ce (4+)**	Powder	0.1–0.5	○ **In vitro cytocompatibility** with MG63 (Ce-HA-NPs at doses in the range **200–600 µg mL^−1^**); ○ Increase of MG-63 **cell viability, proliferation and differentiation** at doses of **200–400 µg mL^−1^**; ○ **Antibacterial effect** against *S. aureus* (ATCC 6538), *Lactobacillus* (ATCC 393), *Bacillus subtilis*, *E. coli* (714), and *P. aeruginosa*; ○ Significant decrease of bacteria number when coupled with Fe_3_O_4_ NPs.	[348,381,385]
**Sm**	Powder Coating	0.2–0.5	○ **In vitro cytocompatibility** with HFOB 1.19 cell line (comparable to pure HA control specimens); ○ **Antibacterial effect** against *S. aureus*, *E. faecalis*, *E. coli*, and *P. aeruginosa*. Differences in the extent of antibacterial activity for Gram-positive and Gram-negative stains; ○ **Antifungal effect** against *C. albicans* (ATCC 10231).	[355,357]
**Eu**	Powder	0.1–20	○ Induces the in vitro formation of bone-like apatite in SBF; ○ **In vitro cytocompatibility** with *MG-63* (cell proliferation up to 4 days), *HeLa*, *human embryonic kidney HEK 293*, *L929* (viability >80% for Eu-HA doses of **25–500 µg mL^−1^**); ○ **Low cytotoxicity** for *human gingival fibroblast (HGF-1)* cells after 24 h (**500–2000 µg mL^−1^**); ○ **Cytotoxicity** for *transformed human umbilical vein endothelial cells (T-HUVEC)* after treatment with at **0.3–30 µg mL^−1^** **of 5 at.% doped HA**; ○ **Ability to kill** *cervical HeLa cells* after 24 when combined with 5 fluorouracil (5FU); ○ **Negligible toxicity by hen’s egg test** on the chick area vasculosa (HET-CAV); ○ **Antibacterial effect** against *E. faecalis* (ATCC 29212), *S. aureus* (0364), and *P. aeruginosa* (1397); **No antibacterial** activity against *E. coli* even at high doping; ○ **Antifungal effect** against *C. albicans* (ATCC 10231) with only for a doping content of **20 at.%**.	[361,362,363,364,365,366,388,389,390,391,392,393]
**Tb**	Powder	2–17	○ **In vitro cytocompatibility** with *MC3T3-E1* (doses of **25–100 µg mL^−1^** Tb-HA-NPs) and A549 (doses of **20–320 µg mL^−1^** Tb-HA-NPs) cell lines.	[361,393,399]
**Gd**	Powder	1–17	○ **In vitro cytocompatibility** with *HFOB 1.19* for x_Gd_ < 17% [Ca_10-x_Gd_x_(PO_4_)_6_(OH)_2_] cell line; ○ **Cytotoxicity for** HFOB 1.19 cells at x_Gd_ ~ 17%.	[128]
**Dy**	Powder	0.5–10	○ **In vitro cytocompatibility** with *L929* cell line; ○ **Negligible toxicity by hen’s egg test** on the chick area vasculosa (HET-CAV); ○ **Increase of oxidative stress** *lipoperoxides* and *nitric oxide* **indicators** in kidney, lungs and liver of rats; **lower activity of anti-oxidant** glutathione peroxidase enzyme.	[392,403]
**Nd**	Powder	1–17	○ **In vitro cytocompatibility** with HFOB 1.19 (for doping concentrations **of 1–17 at.%**) and L929 (cell viability at 24 h > 90% for doses of Nd-HA of **10** and **20 mg mL^−1^**) cell lines.	[128,352]
**Er**	Powder	2–10	○ Induces the formation of biomimetic apatite in-growths in SBF.	[372]
**U**	Solution	0.1–10	○ **In vitro cytocompatibility** with MC3T3-E1 cell line (not sensitive to the presence of uranium).	[375]
